# Loss of Fas signaling in fibroblasts impairs homeostatic fibrosis resolution and promotes persistent pulmonary fibrosis

**DOI:** 10.1172/jci.insight.141618

**Published:** 2020-12-08

**Authors:** Elizabeth F. Redente, Sangeeta Chakraborty, Satria Sajuthi, Bart P. Black, Ben L. Edelman, Max A. Seibold, David W.H. Riches

**Affiliations:** 1 Program in Cell Biology, Department of Pediatrics, National Jewish Health, Denver, Colorado, USA.; 2 Division of Pulmonary Sciences and Critical Care Medicine, Department of Medicine, University of Colorado School of Medicine, Aurora, Colorado, USA.; 3 Department of Research, Veterans Affairs Eastern Colorado Health Care System, Denver, Colorado, USA.; 4 Center for Genes, Environment and Health, National Jewish Health, Denver, Colorado, USA.; 5 Department of Immunology and Microbiology, University of Colorado School of Medicine, Aurora, Colorado, USA.

**Keywords:** Pulmonology, Apoptosis, Fas signaling, Fibrosis

## Abstract

Idiopathic pulmonary fibrosis (IPF) is a progressive, irreversible fibrotic disease of the distal lung alveoli that culminates in respiratory failure and reduced lifespan. Unlike normal lung repair in response to injury, IPF is associated with the accumulation and persistence of fibroblasts and myofibroblasts, as well as continued production of collagen and other extracellular matrix (ECM) components. Prior in vitro studies have led to the hypothesis that the development of resistance to Fas-induced apoptosis by lung fibroblasts and myofibroblasts contributes to their accumulation in the distal lung tissues of IPF patients. Here, we test this hypothesis in vivo in the resolving model of bleomycin-induced pulmonary fibrosis in mice. Using genetic loss-of-function approaches to inhibit Fas signaling in fibroblasts, potentially novel flow cytometry strategies to quantify lung fibroblast subsets, and transcriptional profiling of lung fibroblasts by bulk and single cell RNA sequencing, we show that Fas is necessary for lung fibroblast apoptosis during homeostatic resolution of bleomycin-induced pulmonary fibrosis in vivo. Furthermore, we show that loss of Fas signaling leads to the persistence and continued profibrotic functions of lung fibroblasts. Our studies provide insights into the mechanisms that contribute to fibroblast survival, persistence, and continued ECM deposition in the context of IPF and how failure to undergo Fas-induced apoptosis impairs fibrosis resolution.

## Graphical abstract

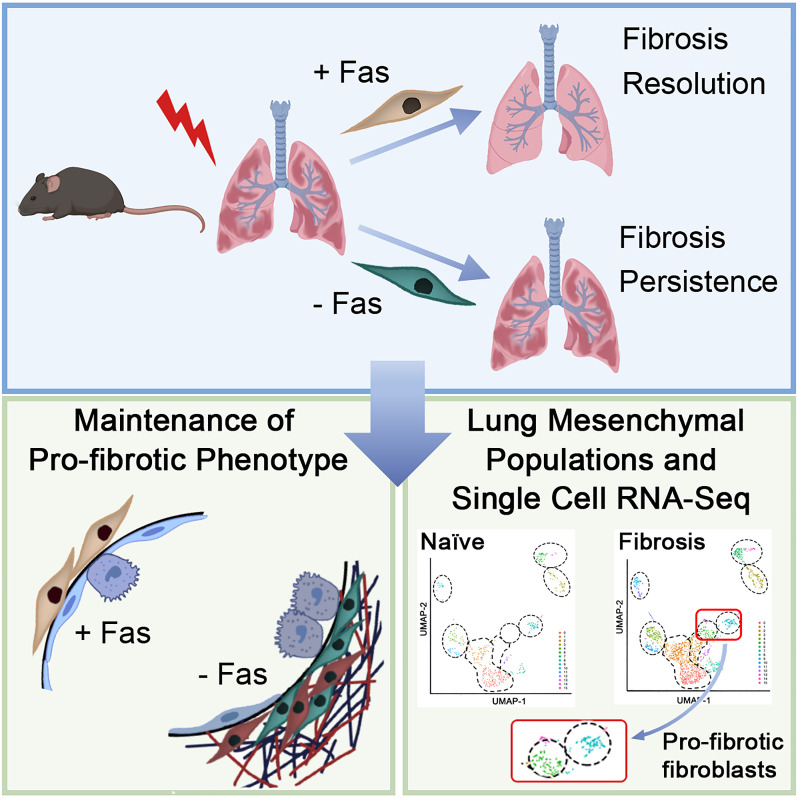

## Introduction

Idiopathic pulmonary fibrosis (IPF), a progressive fibrotic lung disorder of high morbidity and mortality, develops in response to initial alveolar epithelial injury and results in an aberrant repair response in which resident lung interstitial and perivascular fibroblasts proliferate, differentiate into myofibroblasts, and persist within the collapsed alveoli and septal interstitium (1, 2). These fibroblasts and myofibroblasts (collectively referred to herein as profibrotic fibroblasts), continue to produce excessive amounts of extracellular matrix (ECM), which in turn leads to relentless fibrosis, progressive declines in gas exchange, and eventual respiratory failure (1, 2). Acute respiratory distress syndrome (ARDS) patients also exhibit high morbidity and mortality resulting from initial alveolar epithelial injury (3). Likewise, they also accumulate profibrotic fibroblasts that secrete collagen and ECM components within the injured alveoli early in their clinical course (4–6). However, while both conditions share some similarities in the development of the early response to injury, the clinical outcomes of these 2 disorders are substantially different. IPF patients develop a persistent and progressive fibrosis considered by many to be irreversible (1). By contrast, while ARDS patients experience high initial mortality, the early fibroproliferative response can resolve, and lung structure and function can return toward normality in some, though not all, survivors (3, 7, 8).

Little is known about the factors that distinguish these different outcomes in IPF patients and ARDS survivors. In general, fibrosis is considered to be detrimental and is thought to lead to poor clinical outcomes. However, the early fibrosis that develops in ARDS patients could be viewed as a beneficial response in which the provisional ECM scaffold produced by proliferating profibrotic fibroblasts supports the regenerating alveolar epithelium and endothelium. As alveolar structure and function are restored, the majority of the profibrotic fibroblasts undergo apoptosis and clearance (9, 10). During this period of tissue regeneration, macrophages and other cells are thought to degrade and phagocytose the fibrotic provisional ECM scaffold through an integrated process that we define herein as homeostatic fibrosis resolution. In contrast to this trophic regenerative process, the persistent and progressive pulmonary fibrosis that defines IPF patients can be viewed as a failure in homeostatic fibrosis resolution. We hypothesize that a failure in profibrotic lung fibroblast apoptosis and clearance plays a fundamentally important role in impeding homeostatic fibrosis resolution in IPF.

Previous studies by ourselves and others have provided insights into the mechanisms that control lung fibroblast apoptosis. In particular, susceptibility to apoptosis induction by the death receptor Fas has been shown to play a key role (11–16). Fas is expressed by lung fibroblasts, and once a predefined threshold has been exceeded, Fas ligation induces apoptosis (12, 17). In contrast, lung fibroblasts from IPF patients exhibit variable resistance to Fas-induced apoptosis (13, 15) due to downregulation of Fas expression (14, 17, 18) and increased expression of multiple antiapoptotic genes, including Bcl-2, XIAP, cFLIP_L_, and PTPN13 (11, 19–21). Together, these in vitro studies have fueled the concept that the acquired resistance of lung fibroblasts to Fas-induced apoptosis promotes profibrotic fibroblast accumulation in the persistently fibrotic lungs of IPF patients (11, 15, 16, 20, 22, 23). However, until now, this concept has not been directly addressed in vivo.

To address this knowledge gap, we genetically deleted Fas in fibroblasts and investigated the functional consequences on the normally resolving model of bleomycin-induced fibrosis. Intratracheal instillation of a single dose of bleomycin induces inflammatory epithelial injury, followed by a resolving fibrotic response accompanied by regeneration of the distal lung architecture (9, 24). Thus, the bleomycin model recapitulates acute lung injury and early fibrosis, followed by fibrosis resolution and lung regeneration (25), and represents an authentic model of homeostatic fibrosis resolution at later time points. As we will show, loss of Fas signaling in profibrotic fibroblasts impairs homeostatic fibrosis resolution and results in fibroblast persistence in lung tissues along with persistent pulmonary fibrosis, replicating a central feature of IPF. Furthermore, Col1a1- and α-smooth muscle actin–reporter (α-SMA–reporter) mice and bulk and single cell RNA sequencing (scRNA-seq) assessments of purified lung fibroblasts showed that Fas deficiency in fibroblasts results in preserved patterns of increased ECM and fibrotic gene expression in the persistently fibrotic lungs. We conclude that Fas-induced fibroblast apoptosis plays a necessary role in fibroblast elimination during homeostatic fibroblast resolution, and that resistance to Fas-mediated apoptosis promotes persistent pulmonary fibrosis.

## Results

### Fas deletion in fibroblasts impairs lung homeostatic fibrosis resolution.

**Figure 1:**
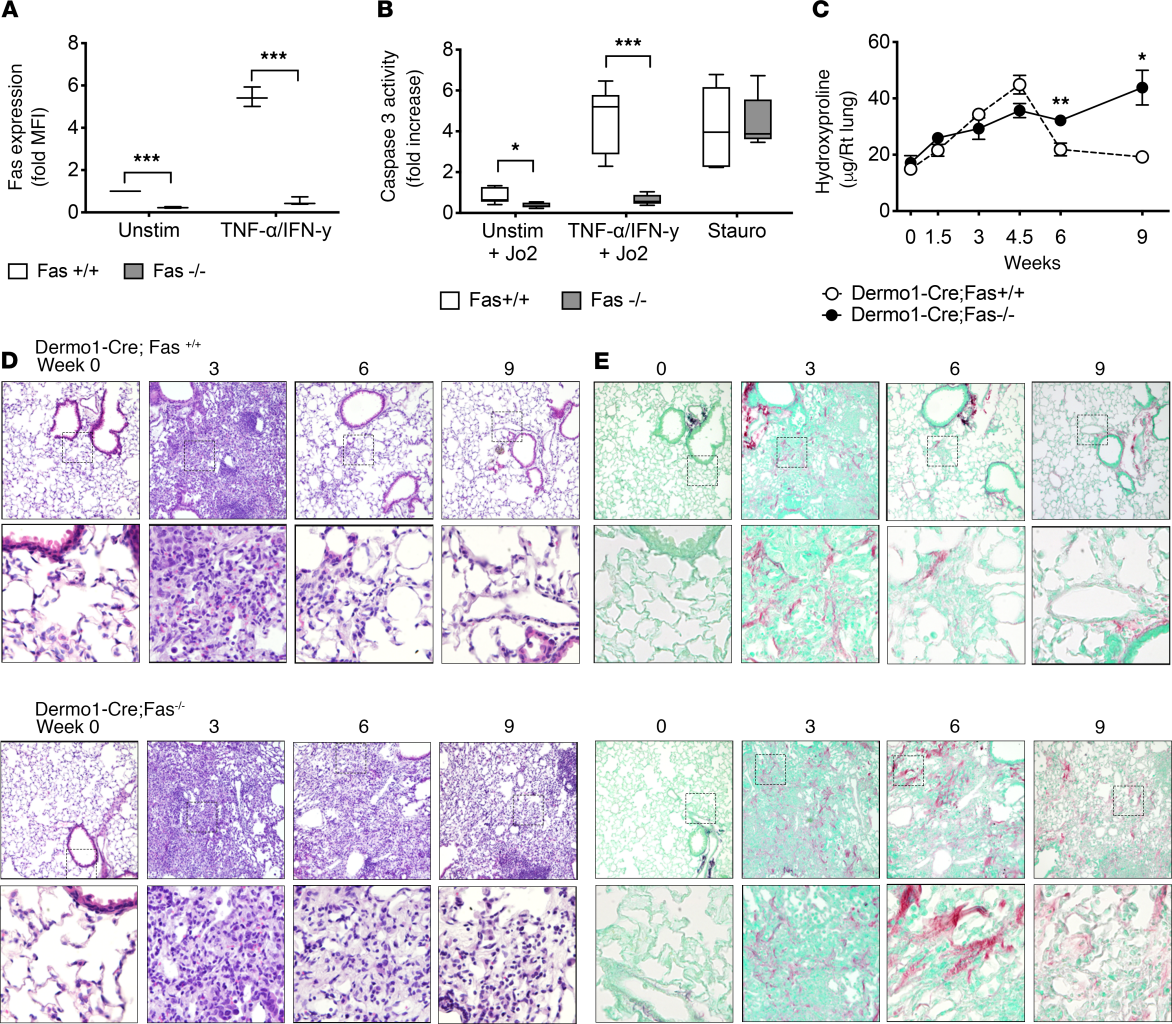
Fas expression in mesenchymal cells is essential for their spontaneous apoptosis during fibrosis resolution. (**A**) Cell surface expression on cultured fibroblasts from WT and Fas-deficient mice. (**B**) Caspase 3 activity in WT and Fas-deficient fibroblasts after Fas ligation. (**C**) Hydroxyproline levels in the lungs over time after bleomycin in Dermo1-Cre;Fas^+/+^ and Dermo1-Cre;Fas^–/–^ mice. (**D** and **E**) Representative H&E-stained (**D**) and Picrosirius red–stained (**E**) lung sections over time in Dermo1-Cre;Fas^+/+^ and Dermo1-Cre;Fas^–/–^ mice. Box-and-whisker plots show median, minimum, and maximum values. Time course is mean ± SEM, *n* = 6–8. *P < 0.05, **P < 0.01, 2-tailed *t* test with Welch’s correction. Total magnification, 200× (upper panels) and 400× (lower panels).

To investigate the functional role of Fas expression by fibroblasts in homeostatic fibrosis resolution, we deleted Fas in mesenchymal cells by breeding *Fas^fl/fl^* mice with *Dermo1(Twist2)-Cre* mice (26) to create *Dermo1-Cre;Fas^fl/fl^* mice (abbreviated as Dermo1-Cre;Fas^–/–^). To ensure that Fas was deleted, we isolated lung fibroblasts from WT and Dermo1-Cre;Fas^–/–^ mice, and we assessed their level of cell surface Fas expression by flow cytometry and their ability to undergo apoptosis (a) following sensitization with TNF-α and IFN-α and Fas-ligation with agonistic anti-Fas antibody (Jo2), and (b) in response to staurosporine, an activator of the intrinsic apoptosis pathway. In comparison with WT lung fibroblasts, fibroblasts from Dermo1-Cre;Fas^–/–^ mice displayed minimal cell surface Fas basally or in response to stimulation with TNF-α and IFN-α (Figure 1A) and were not susceptible to Fas-induced apoptosis (Figure 1B). However, fibroblasts from both WT and Dermo1-Cre;Fas^–/–^ mice showed similar levels of apoptosis induction in response to staurosporine (Figure 1B). Together, these data indicate that in vivo genetic deletion of Fas in mesenchymal cells led to loss of cell surface Fas and corresponding apoptosis induction by the Fas-induced extrinsic apoptosis pathway, but not by staurosporine.

Next, we evaluated the ability of Dermo1-Cre;Fas^–/–^ mice to undergo homeostatic fibrosis resolution in response to a single intratracheal instillation of bleomycin (1.5 U/kg). WT mice exhibit a predictable course of acute inflammatory lung injury at 1 week, fibrosis development at 2–4 weeks, and resolution to near-normal lung structure and function by 6–9 weeks (24). Dermo1-Cre;Fas^–/–^ and control Dermo1-Cre;Fas^+/+^ mice developed similar amounts of bleomycin-induced pulmonary fibrosis, as reflected by lung hydroxyproline levels at 1.5, 3, and 4.5 weeks (Figure 1C). Both strains also exhibited similar changes in lung histopathology and patterns of peribronchiolar and parenchymal collagen deposition, as revealed by Picrosirius red staining (PRS) (Figure 1, D and E). However, unlike Dermo1-Cre;Fas^+/+^ mice, which had undergone homeostatic fibrosis resolution at 6 and 9 weeks, the lungs of Dermo1-Cre;Fas^–/–^ mice showed persistent fibrosis and histopathologic distortion at 6 and 9 weeks (Figure 1, C–E). WT C57BL/6 mice and Dermo1-Cre;Fas^+/+^ mice showed identical patterns of fibrosis development and homeostatic fibrosis resolution (data not shown).

**Figure 2:**
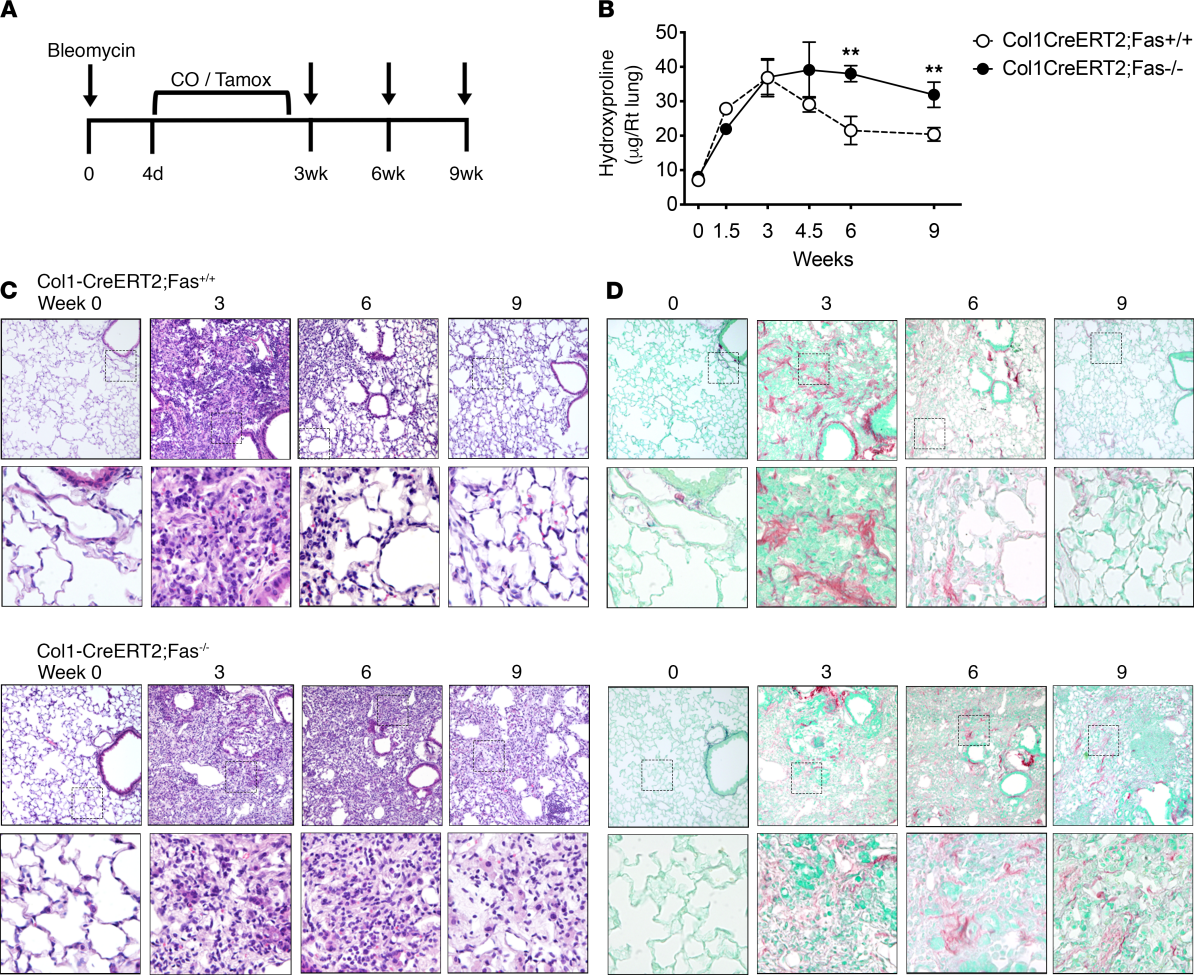
Fas expression in collagen producing cells is essential for their spontaneous apoptosis during fibrosis resolution. (**A**) Sketch illustrating instillation time, tamoxifen dosing, and harvest time points in Col1-CreERT2;Fas^fl/fl^ mice. (**B**) Hydroxyproline levels in the lungs over time after bleomycin in Col1-CreERT2;Fas^+/+^ and Col1-CreERT2;Fas^–/–^ mice. Representative H&E-stained (**C**) and Picrosirius red–stained (**D**) lung sections over time in Col1-CreERT2;Fas^+/+^ and Col1-CreERT2;Fas^–/–^ mice. Box-and-whisker plots show median, minimum and maximum values. Time course is mean ± SEM, *n* = 6-8. **P < 0.01, 2-tailed *t* test with Welch’s correction. Total original magnification, 200× (upper panels) and 400× (lower panels).

Profibrotic fibroblasts express multiple ECM genes, including Col1a1 (Supplemental Figure 1; supplemental material available online with this article; https://doi.org/10.1172/jci.insight.141618DS1). We therefore determined if conditional deletion of Fas in Col1a1-expressing cells phenocopied the impairment of homeostatic fibrosis resolution seen in Dermo1-Cre;Fas^–/–^ mice. *Fas^fl/fl^* mice were bred with *Col1a1-CreERT2* mice (27) to create *Col1a1-Cre-ERT2;Fas^fl/fl^* mice, which we refer to as Col1-CreERT2;Fas^–/–^ following tamoxifen injection, and Col1-CreERT2;Fas^+/+^ following corn oil vehicle injection. As illustrated in Figure 2A, *Col1-CreERT2;Fas^fl/fl^* mice were instilled with bleomycin, treated with either tamoxifen or corn oil twice weekly during fibrosis development between weeks 0.5 and 3, and assessed for fibrotic outcomes between weeks 1.5 and 9. Recombination at the *Fas^fl/fl^* locus in tamoxifen-treated mice was confirmed by PCR analysis of tail tip fibroblast DNA (Supplemental Figure 2). Figure 2B shows that tamoxifen-injected Col1-CreERT2;Fas^–/–^ mice and corn oil–injected Col1-CreERT2;Fas^+/+^ mice developed similar amounts of fibrosis at 1.5 and 3 weeks. However, whereas bleomycin-instilled, Col1-CreERT2;Fas^+/+^ mice underwent homeostatic fibrosis resolution between 4.5 and 9 weeks, homeostatic fibrosis resolution was impaired in the lungs of bleomycin-instilled Col1-CreERT2;Fas^–/–^ mice, as reflected by persistently elevated lung hydroxyproline levels (Figure 2B) and sustained fibrotic lung histology patterns (Figure 2, C and D). WT C57BL/6 mice and Col1-CreERT2;Fas^+/+^ mice showed indistinguishable patterns of fibrosis development and homeostatic fibrosis resolution (ref. 24 and data not shown). Together, these data show that loss of Fas signaling in Col1a1-expressing profibrotic fibroblasts also led to impaired homeostatic fibrosis resolution and persistent pulmonary fibrosis.

### Fas deletion in fibroblasts promotes lung fibroblast persistence.

**Figure 3:**
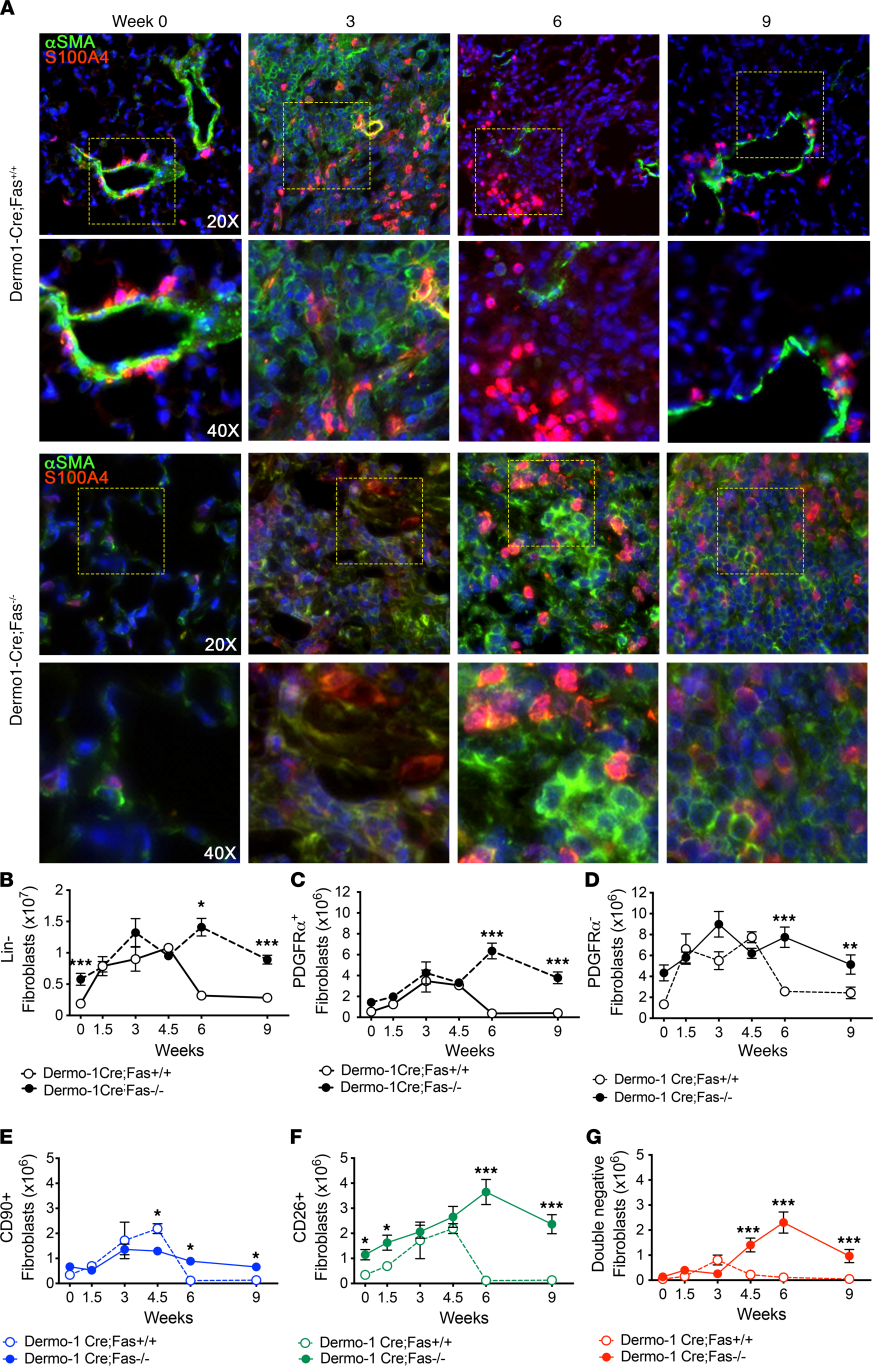
Fibroblasts persist in fibrotic lungs in the absence of Fas. (**A**) α-SMA (green) and S100A4 (red) immunofluorescence staining of lungs over time after bleomycin in Dermo1-Cre;Fas^+/+^ and Dermo1-Cre;Fas^–/–^ mice. (**B**–**D**) Quantification of Lin^–^, PDGFRα^+^, and PDGFRα^–^ fibroblasts populations. (**E**–**G**) Quantification of CD90^+^CD26^–^, CD26^+^CD90^–^, and CD90^–^CD26^–^ fibroblast subsets. Time course is mean ± SEM, *n* = 6–8. *P < 0.05, **P < 0.01, ***P < 0.001, 2-tailed *t* test with Welch’s correction. Total original magnification, 200× (upper panels) and 400× (lower panels).

We next assessed the consequences of loss of Fas signaling on lung fibroblast numbers. Dermo1-Cre;Fas^–/–^ and Dermo1-Cre;Fas^+/+^ mice were instilled with saline or bleomycin, and lung tissues were harvested at 3, 6, and 9 weeks. Lung fibroblasts were initially identified in lung paraffin sections by Immunofluorescence staining for α-SMA and S100A4 (aka FSP1). Figure 3A shows that, while naive lung tissues exhibited minimal staining for S100A4 and α-SMA in the distal alveoli, bleomycin-instilled Dermo1-Cre;Fas^–/–^ mice and Dermo1-Cre;Fas^+/+^ mice exhibited abundant and concordant staining of α-SMA^+^, S100A4^+^, and α-SMA^+^S100A4^+^ fibroblasts in the fibrotic lung parenchyma at 3 weeks. However, whereas parenchymal α-SMA and S100A4 staining was reduced at 6 and 9 weeks in the regenerated lung parenchyma of Dermo1-Cre;Fas^+/+^ mice, α-SMA^+^, S100A4^+^, and α-SMA^+^S100A4^+^ fibroblasts remained in the persistently fibrotic lungs of Dermo1-Cre;Fas^–/–^ mice at 6 and 9 weeks (Figure 3A).

To confirm these findings, we used enzymatic lung cell dispersal and multiparameter flow cytometry to assess the consequences of loss of Fas signaling on lung fibroblast numbers in Dermo1-Cre–expressing cells. Lung single cell suspensions were analyzed using the gating strategy shown in Supplemental Figure 3A and Supplemental Table 1. From these data, we quantified the number of lineage^–^ (Lin^–^) cells, which are thought to be primarily fibroblasts (28–30), by excluding EpCAM/CD326^+^ (epithelial), CD45^+^ (hematopoietic), and CD31^+^ (endothelial) populations. Dermo1-Cre;Fas^+/+^ and Dermo1-Cre;Fas^–/–^ mice were instilled with saline or bleomycin, and the numbers of Lin^–^ cells quantified for up to 9 weeks. Figure 3B shows that, during the development of fibrosis between 1.5 and 3 weeks, the number of Lin^–^ lung fibroblasts increased approximately 2.3-fold, with similar numbers being observed in both Dermo1-Cre;Fas^+/+^ and Dermo1-Cre;Fas^–/–^ mice. Within the Lin^–^ fraction, approximately 33% expressed variable levels of the pan-fibroblast marker PDGFRα, while 66% were PDGFRα^–^. Figure 3, B–D, also shows that, whereas the numbers of total lung Lin^–^ cells, Lin^–^PDGFRα^+^, and Lin^–^PDGFRα^–^ cells in Dermo1-Cre;Fas^+/+^ mice were progressively reduced by 6 and 9 weeks, their numbers remained elevated at 6 and 9 weeks in Dermo1-Cre;Fas^–/–^ mice.

Novel strategies to identify pulmonary fibroblast subsets by flow cytometry are still needed. Therefore, we sought to utilize cell surface markers to identify fibroblasts and segregate them into function-related subsets using PDGFRα (a pan fibroblast marker), CD90 (Thy1), and CD26 (Dpp4). CD90 (Thy1) is expressed by lipid-laden resident alveolar interstitial fibroblasts and perivascular pericyte-like cells (31–34), while CD26 is expressed by matrix-producing reticular dermal fibroblasts (35). Three Lin^–^ subsets that express variable levels of PDGFRα were identified: CD90^+^CD26^–^, CD90^–^CD26^+^, and CD90^–^CD26^–^, which we abbreviate as CD90^+^, CD26^+^, and CD90^–^CD26^–^ subsets (Supplemental Figure 3A), respectively. We confirmed the presence of CD90 and CD26 in lung fibroblasts by flow cytometry of dispersed lung tissues from saline- and bleomycin-instilled C57BL/6 mice at 3 weeks (Supplemental Figure 3A) (28, 36–38). CD90^+^ fibroblasts were the most abundant subset in the lungs of naive and saline-instilled C57BL/6 mice in comparison with CD26^+^ cells and CD90^–^CD26^–^ cells (Supplemental Figure 3, B–D). However, whereas the numbers of CD90^+^ fibroblasts were only modestly increased (1.2-fold) following bleomycin instillation, CD26^+^ and CD90^–^CD26^–^ fibroblasts underwent a large expansion (7.7-fold and 9.6-fold, respectively) compared with saline-instilled mice at 3 weeks (Supplemental Figure 3, B–D).

We next assessed the consequences of Fas deletion in fibroblasts on the numbers of CD90^+^, CD26^+^, and CD90^–^CD26^–^ fibroblasts in bleomycin-instilled Dermo1-Cre;Fas^–/–^ and Dermo1-Cre;Fas^+/+^ mice. Figure 3, E–G, and Supplemental Figure 4, A and B, show that — following bleomycin instillation — the numbers of CD90^+^, CD26^+^, and CD90^–^CD26^–^ fibroblasts expanded as fibrosis developed in Dermo1-Cre;Fas^+/+^ mice, peaking between 1.5 and 4.5 weeks before declining to baseline as the mice underwent homeostatic fibrosis resolution by 6 and 9 weeks. Interestingly, the decline in fibroblast numbers was slightly delayed when compared with the rate of decline in lung collagen levels (Figure 1C). Lung CD90^+^, CD26^+^, and CD90^–^CD26^–^ fibroblast numbers also increased by 3 weeks in Dermo1-Cre;Fas^–/–^ mice (Figure 3, E–G, and Supplemental Figure 4, A and B). However, in contrast to Dermo1-Cre;Fas^+/+^ mice, their numbers remained elevated at 4.5, 6, and 9 weeks (Figure 3, E–G, ).

**Figure 4:**
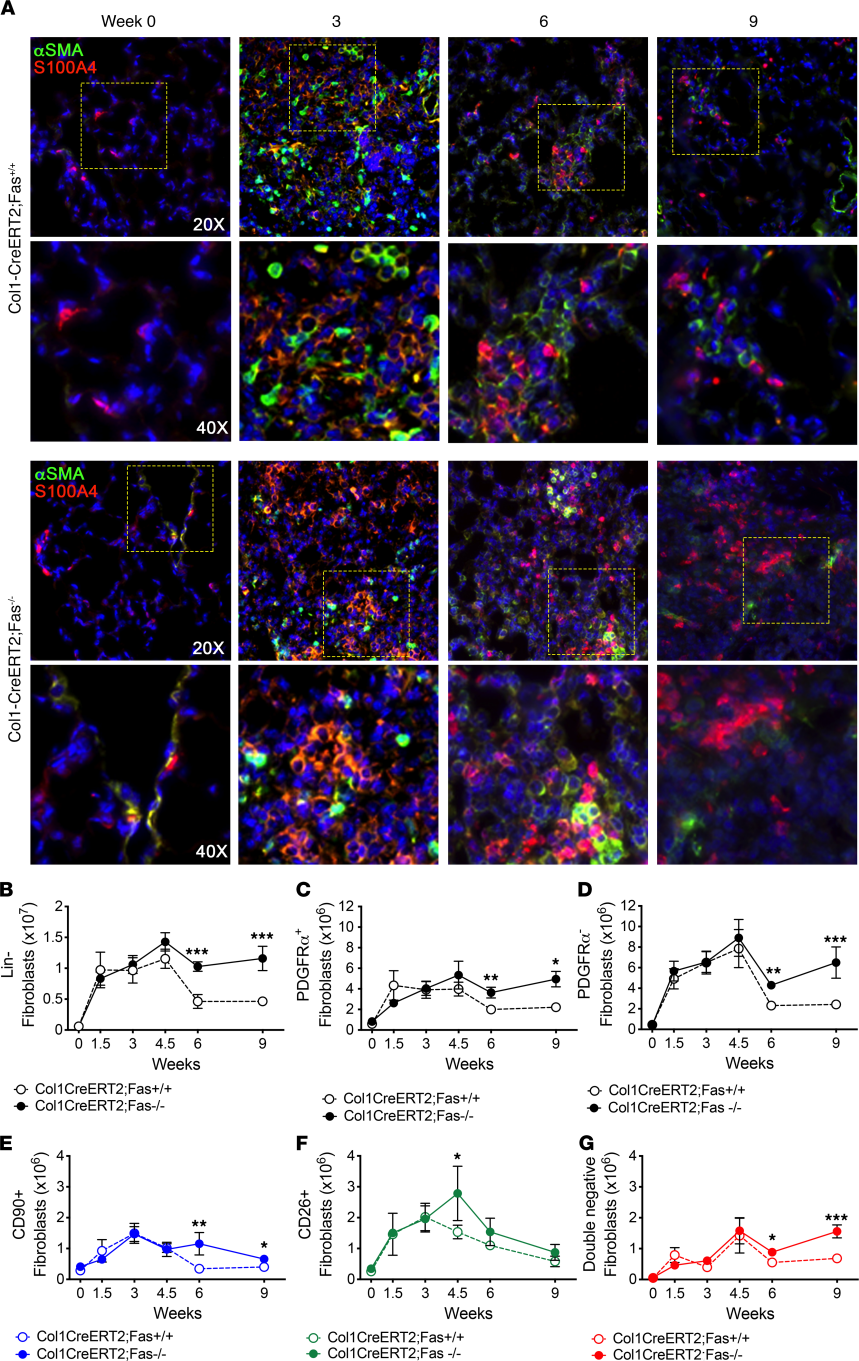
Deletion of Fas in Col-1 fibroblasts permits their persistence during fibrosis. (**A**) α-SMA (green) and S100A4 (red) immunofluorescence staining of lungs over time after bleomycin in Col1-CreERT2;Fas^+/+^ and Col1-CreERT2;Fas^–/–^ mice. (**B**–**D**) Quantification of Lin^–^, PDGFRα^+^ and PDGFRα^–^ fibroblast populations. (**E**–**G**) Quantification of CD90^+^CD26^–^, CD26^+^CD90^–^, and CD90^–^CD26^–^ fibroblast subsets. Time course is mean ± SEM, *n* = 6–9. *P < 0.05, **P < 0.01, ***P < 0.001, 2-tailed *t* test with Welch’s correction. Total original magnification, 200× (upper panels) and 400× (lower panels).

To confirm these findings, *Col1-CreERT2;Fas^fl/fl^* mice were instilled with saline or bleomycin and injected with tamoxifen or corn oil between weeks 0.5 and 3 (Figure 2A), and lung tissues were harvested at 3, 6, and 9 weeks. As was seen with the Dermo1-Cre;Fas^+/+^ and Dermo1-Cre;Fas^–/–^, bleomycin-instilled Col1-CreERT2;Fas^–/–^ mice and Col1-CreERT2;Fas^+/+^ mice exhibited similar staining of α-SMA^+^, S100A4^+^, and α-SMA^+^S100A4^+^ fibroblasts at 3 weeks (Figure 4A). However, whereas peripheral α-SMA and S100A4 staining was reduced at 6 and 9 weeks in the regenerated lung parenchyma of Col1-CreERT2;Fas^+/+^ mice and α-SMA^+^, S100A4^+^, and α-SMA^+^S100A4^+^ fibroblasts were still detected in lungs of Col1-CreERT2;Fas^–/–^ mice at 6 and 9 weeks (Figure 4A). Similarly, flow cytometry revealed a similar approximately 2-fold increase in Lin^–^ fibroblast numbers in both Col1-CreERT2;Fas^–/–^ and Col1-CreERT2;Fas^+/+^ mice during fibrosis development between 1.5 and 3 weeks (Figure 4B) and within the Lin^–^ fraction, approximately 35% expressed variable levels of PDGFRα, while 65% were PDGFRα^–^ (Figure 4, B–D, and Supplemental Figure 4, C and D). Figure 4, B–D, also shows that, whereas the numbers of total lung Lin^–^ cells, Lin-PDGFRα^+^, and Lin-PDGFRα^–^ cells in Col1-CreERT2;Fas^+/+^ declined toward baseline by 6 and 9 weeks, their numbers remained elevated at 6 and 9 weeks in Col1-CreERT2;Fas^–/–^ mice. Lung CD90^+^, CD26^+^, and CD90^–^CD26^–^ fibroblast numbers also increased between 1.5 and 4.5 weeks in Col1-CreERT2;Fas^–/–^ mice, but — in contrast to Col1-CreERT2;Fas^+/+^ mice — their numbers remained significantly elevated at 6 and 9 weeks (Figure 4, E–G). To affirm the specificity of the Col1a1 promoter–driven Fas deletion strategy, we lineage traced with TdTomato. Supplemental Figure 5 shows that TdTomato was primarily expressed in Lin^–^PDGFRα^+^ cells. Thus, Fas deficiency in Dermo1-Cre– and Col1a1-expressing fibroblasts impedes fibroblast elimination from lung tissues during homeostatic resolution of bleomycin-induced fibrosis.

### Fas deletion in fibroblasts impairs their apoptosis during homeostatic fibrosis resolution.

**Figure 5:**
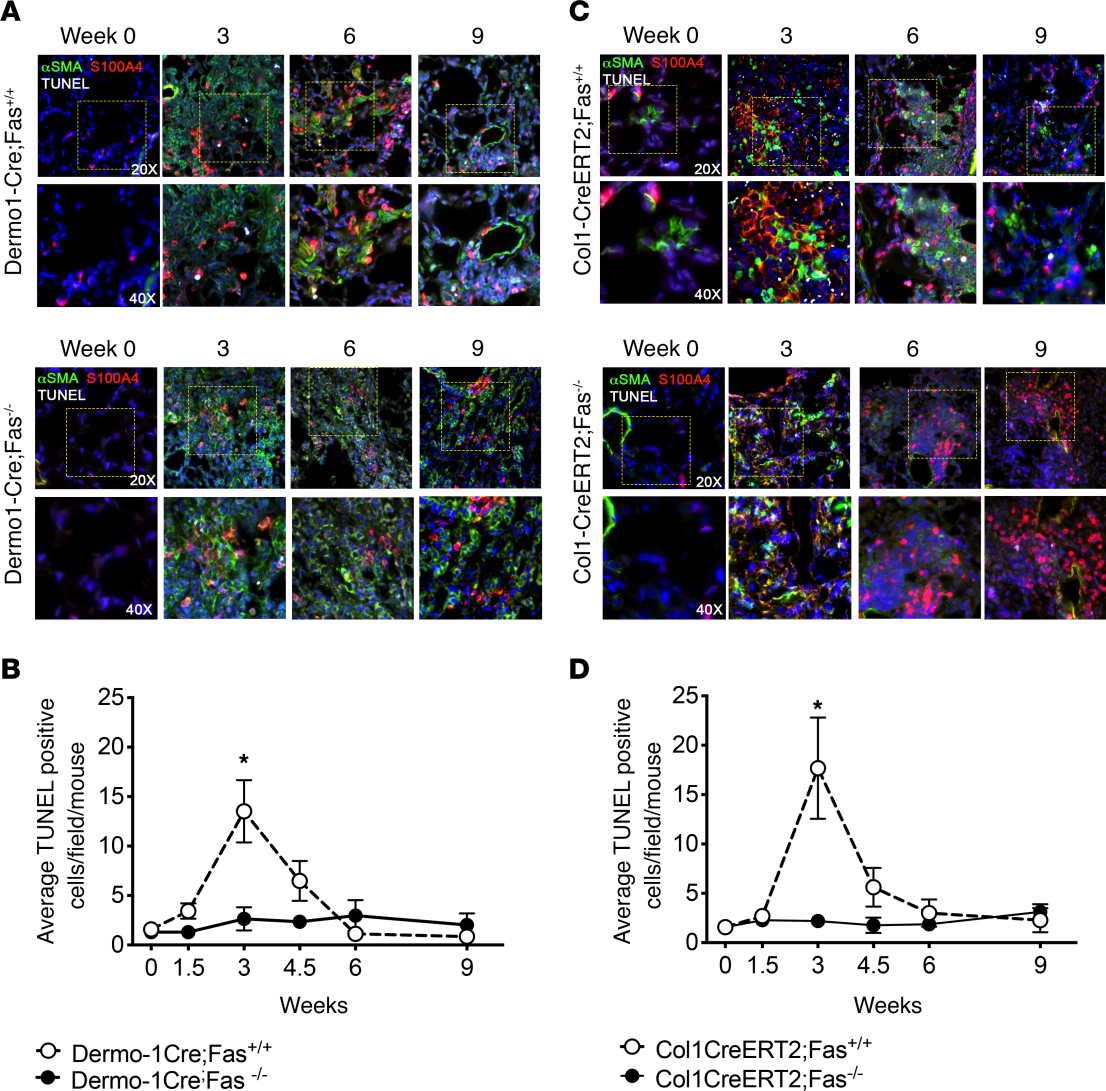
Loss of Fas reduces apoptosis of fibroblasts during fibrosis resolution in vivo. (**A** and **B**) Immunofluorescence staining and quantification of fibroblasts using antibodies for α-SMA (green), S100A4 (red), and TUNEL (white) in Dermo1-Cre;Fas^+/+^ and Dermo1-Cre;Fas^–/–^ mice over time after bleomycin. (**C** and **D**) Immunofluorescence staining and quantification of fibroblasts using antibodies for SMA (green), S100A4 (red), and TUNEL (white) in Col1-CreERT2;Fas^+/+^ and Col1-CreERT2;Fas^–/–^ mice over time after bleomycin. Time course is mean ± SEM, *n* = 10 images per animal/time point. *P < 0.05, 2-tailed *t* test with Welch’s correction. Total original magnification, 200× (upper panels) and 400× (lower panels).

We next investigated the effect of Fas deficiency on lung fibroblast apoptosis in vivo. Dermo1-Cre;Fas^+/+^ and Dermo1-Cre;Fas^–/–^ mice were instilled with saline or bleomycin, and lung tissues were harvested from 1.5–9 weeks. Lung sections were then subjected to triple-label Immunofluorescence staining for TUNEL, α-SMA, and S100A4 (Figure 5A), and TUNEL^+^ fibroblasts were quantified. Figure 5B shows minimal numbers of apoptotic TUNEL^+^ fibroblasts in naive Dermo1-Cre;Fas^+/+^ mice. Increased numbers were first detected in bleomycin-instilled mice at 1.5 weeks, peaked at 3 weeks, and declined to naive numbers at 6 weeks, confirming a previous report (10). Following Fas deletion in fibroblasts in Dermo1-Cre;Fas^–/–^ mice, the number of TUNEL^+^ fibroblasts was not significantly elevated over baseline values at any time point, but when compared with Dermo1-Cre;Fas^+/+^ mice, it significantly reduced at 1.5 weeks (*P* = 0.06), 3 weeks (*P* < 0.05), and 4.5 weeks (*P* = 0.08) (Figure 5, A and B). We confirmed these data in naive and bleomycin-instilled *Col1-CreERT2;Fas^fl/fl^* mice following tamoxifen or corn oil injection between 0.5 and 3 weeks, with harvest and analysis for TUNEL^+^ cells for up to 9 weeks. Figure 5, C and D, shows that Col1-CreERT2;Fas^+/+^ mice also exhibited a peak in TUNEL^+^ apoptotic lung fibroblasts at 3 weeks, while TUNEL^+^ fibroblast numbers were not elevated above baseline at any time point in Col1-CreERT2;Fas^–/–^ mice (*P* = 0.02). Taken together, these data show that Fas deletion in fibroblasts prevents homeostatic fibroblast apoptosis in bleomycin-injured fibrotic lung tissues.

### Fas deficiency in Col1a1-expressing cells leads to persistent Col1a1 and α-SMA promoter activity.

**Figure 6:**
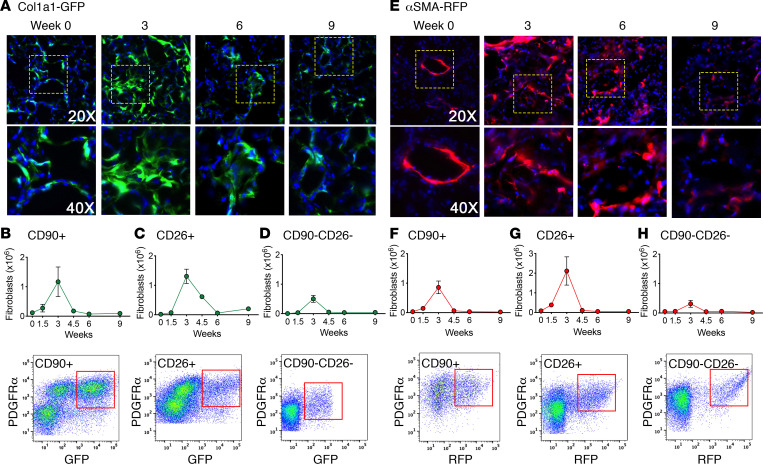
Fibroblast expression of Col1 and α-SMA during fibrosis development and resolution. (**A**) GFP expression of Col1a1 in fibroblasts over time after bleomycin in Col1-GFP mice. (**B**–**D**) Quantification and representative flow cytometry plots of GFP^+^ CD90^+^CD26, CD26^+^CD90^–^, and CD90^–^CD26^–^ fibroblast subsets over time. (**E**) RFP expression of α-SMA in fibroblasts over time after bleomycin in α-SMA–RFP mice. (**F**–**H**) Quantification and representative flow cytometry plots of RPF^+^ CD90^+^CD26^–^, CD26^+^CD90^–^, and CD90^–^CD26^–^ fibroblast subsets over time. Time course is mean ± SEM, *n* = 5–12. Total original magnification, 200× (upper panels) and 400× (lower panels).

Col1-GFP mice express GFP under the control of the Col1a1 promoter (39), while α-SMA–red fluorescent protein (α-SMA–RFP) mice express RFP under the control of the α-SMA promoter (40). We used these mice to determine if the Fas-deficient fibroblasts that remain in persistently fibrotic lung tissues of bleomycin-instilled mice continue to functionally express these profibrotic genes. Frozen lung sections from naive mice revealed modest numbers of dim GFP^+^ fibroblastic interstitial cells, while bleomycin-instilled Col1-GFP mice exhibited increased numbers and intensity of GFP^+^ interstitial cells, peaking at 3 weeks before returning toward baseline by 6–9 weeks (Figure 6A). Flow cytometry analysis of lung single cell suspensions from bleomycin-instilled Col1-GFP mice showed that Lin^–^PDGFRα^+^CD90^+^, CD26^+^, and CD90^–^CD26^–^ fibroblast subsets all exhibited increased GFP expression that peaked at 3 weeks before declining to baseline by 6 weeks (Figure 6, B–D, and Supplemental Figure 6). Naive α-SMA–RFP mice also exhibited minimal RFP expression in the distal lung, though the smooth muscle surrounding arteries and larger airways exhibited well-defined RFP fluorescence (Figure 6E). Bleomycin instillation resulted in increased numbers of interstitial cells expressing RFP in the fibrotic distal lung tissues in lung frozen sections (Figure 6E) and increased numbers of RFP^+^ Lin^–^PDGFRα^+^CD90^+^, CD26^+^, and CD90^–^CD26^–^ fibroblast subsets that peaked at 3 weeks before declining to baseline by 6 weeks (Figure 6, F–H, and Supplemental Figure 7). Neither GFP nor RFP was detected in CD45^+^, CD31^+^, or CD326^+^ cells in bleomycin-instilled Col1-CreERT2;Fas^–/–^;Col1-GFP or Col1-CreERT2;Fas^–/–^;α-SMA–RFP mice, respectively (Supplemental Figure 8). Thus, Col1-GFP and α-SMA–RFP mice quantitatively report changes in profibrotic fibroblast Col1a1 and α-SMA promoter activity, as previously reported (40–44).

**Figure 7:**
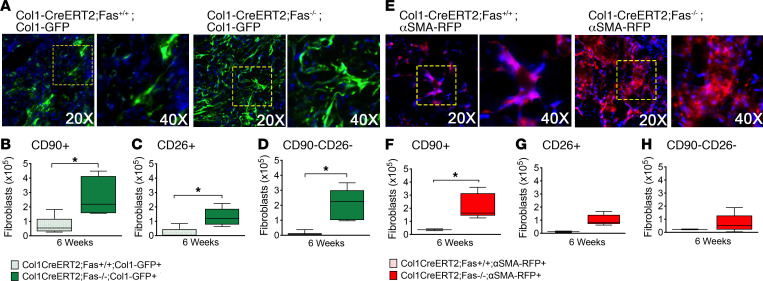
Fibroblast expression of Col1 and α-SMA during fibrosis persistence. (**A**) GFP expression of Col1a1 in fibroblasts 6 weeks after bleomycin in Col1-CreERT2;Fas^+/+^;Col1-GFP and Col1-CreERT2;Fas^–/–^;Col1-GFP mice. (**B**–**D**) Quantification of GPF^+^ CD90^+^CD26^–^, CD26^+^CD90^–^, and CD90^–^CD26^–^ fibroblast subsets. (**E**) RFP expression of α-SMA in fibroblasts 6 weeks after bleomycin in Col1-CreERT2;Fas^+/+^;α-SMA–RFP and Col1-CreERT2;Fas^–/–^;α-SMA–RFP mice. (**F**–**H**) Quantification of RPF^+^ CD90^+^CD26^–^, CD26^+^CD90^–^, and CD90^–^CD26^–^ fibroblast subsets. Box-and-whisker plots show median, minimum, and maximum values. *n* = 5. *P < 0.05, 2-tailed *t* test with Welch’s correction.

To determine if the Fas-deficient fibroblasts that persist in fibrotic lung tissues continue to exhibit Col1a1 promoter activity, we bred *Col1-CreERT2;Fas^fl/fl^* mice with Col1-GFP mice to obtain *Col1-CreER;Fas^fl/fl^;Col1-GFP* mice. *Col1-CreER;Fas^fl/fl^;Col1-GFP* were instilled with bleomycin and injected with tamoxifen or corn oil between 0.5 and 3 weeks to yield Col1-CreERT2;Fas^–/–^;Col1-GFP and Col1-CreERT2;Fas^+/+^;Col1-GFP mice, respectively. The mice were harvested at 6 weeks and analyzed for the presence of GFP in lung fibroblasts in lung frozen sections and by multiparameter flow cytometry. Figure 7A shows that, whereas GFP^+^ fibroblast numbers in frozen sections of bleomycin-instilled Col1-CreERT2;Fas^+/+^;Col1-GFP mice had returned to naive levels at 6 weeks, increased numbers of GFP^+^ cells remained in the lungs of Col1-CreERT2;Fas^–/–^;Col1-GFP. Similarly, flow cytometry of lung single cell suspension revealed increased numbers of GFP^+^ Lin^–^PRGFRα^+^CD90^+^, CD26^+^, and CD90^–^CD26^–^ fibroblasts in the fibrotic lung tissues of Col1-CreERT2;Fas^–/–^;Col1-GFP mice at 6 weeks compared with bleomycin-instilled Col1-CreERT2;Fas^+/+^;Col1-GFP mice (Figure 7, B–D).

We also determined if the Fas-deficient fibroblasts that persist in fibrotic lungs also continued to express α-SMA by breeding *Col1-CreERT2;Fas^fl/fl^* mice with α-SMA–RFP reporter mice using the same strategy as discussed above to generate Col1-CreERT2;Fas^+/+^;α-SMA–RFP mice and Col1-CreERT2;Fas^–/–^;α-SMA–RFP mice. Compared with Fas-sufficient Col1-CreERT2;Fas^+/+^;α-SMA–RFP mice, lung tissues of Col1-CreERT2;Fas^–/–^;α-SMA–RFP mice displayed abundant RFP fluorescence in the fibroblastic cells in the persistently fibrotic distal lung tissues at 6 weeks (Figure 7E). Interestingly, and in contrast to the Col1-CreERT2;Fas^–/–^;Col1-GFP mice, flow cytometry of lung cell suspensions revealed significantly increased numbers of RFP^+^ Lin^–^PRGFRα^+^CD90^+^, but not of CD26^+^ and CD90^–^CD26^–^, fibroblasts in the fibrotic lung tissues of Col1-CreERT2;Fas^–/–^;α-SMA–RFP mice (Figure 7, F–H). Taken together, these data suggest that the Fas-deficient fibroblasts that remain in fibrotic lung tissues of bleomycin-instilled mice at 6 weeks continue to express functional profibrotic Col1a1 and α-SMA promoter activity.

### Bulk and scRNA-seq defines preserved profibrotic fibroblast clusters and signatures in the absence of Fas signaling.

**Figure 8:**
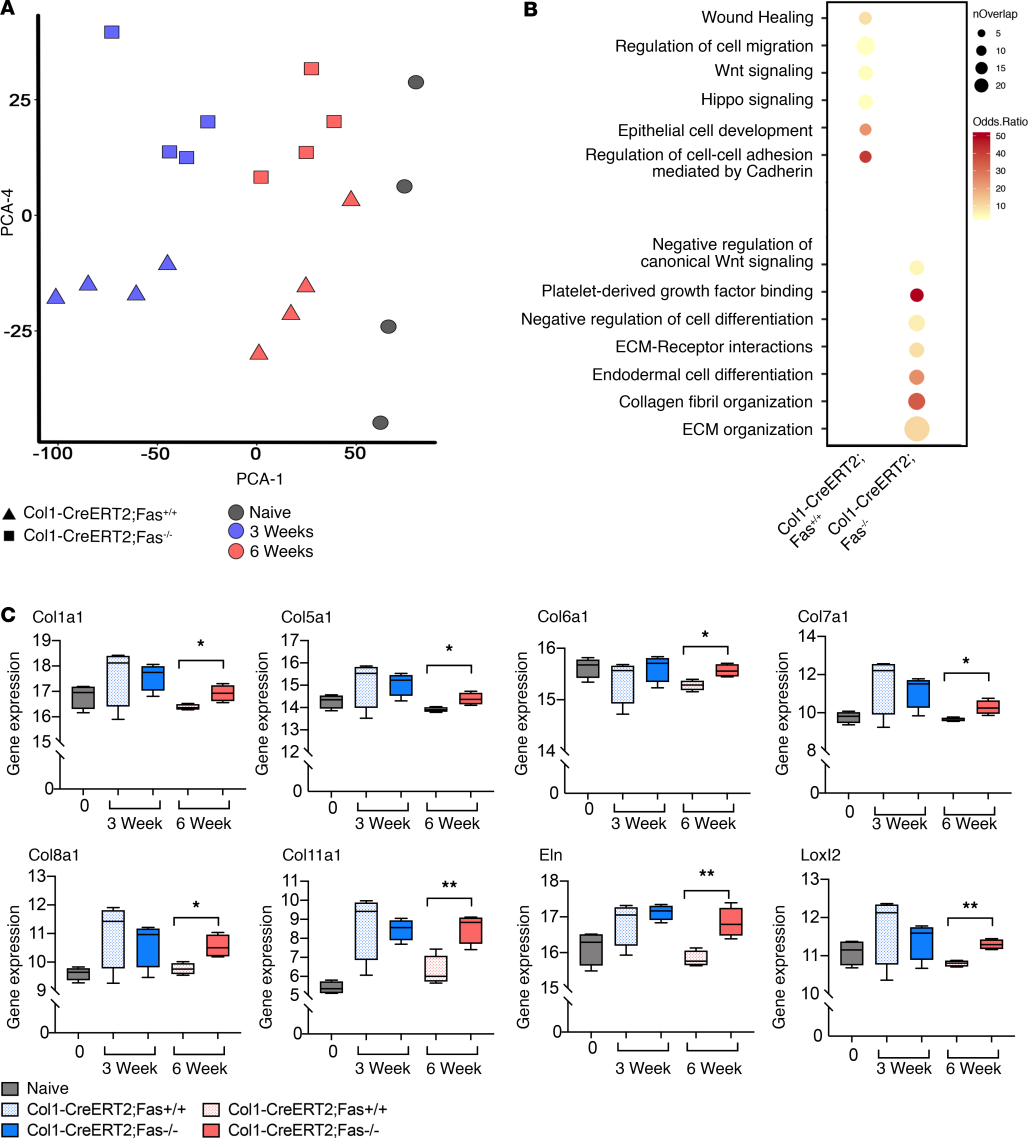
Bulk sequencing reveals a sustained profibrotic signature in fibroblasts in the absence of Fas. (**A**) PCA plot of global transcription patterns in Col1-CreERT2;Fas^+/+^ and Col1-CreERT2;Fas^–/–^ Lin^–^ fibroblasts from control and bleomycin-treated mice after 3 and 6 weeks. (**B**) Representation of enrichment for GO pathway categories 6 weeks after bleomycin from Col1-CreERT2;Fas^+/+^ and Col1-CreERT2;Fas^–/–^ Lin^–^ fibroblasts. (**C**) Box-and-whisker plots of genes expressed during fibrosis and in nonresolving Col1-CreERT2;Fas^–/–^ fibroblasts. Box-and-whisker plots show median, minimum, and maximum values. *n* = 4. *P < 0.05, **P < 0.01, 2-tailed *t* test with Welch’s correction.

To further characterize the fibroblasts that persist in fibrotic lung tissues in the absence of Fas signaling, we conducted bulk and scRNA-seq on sorted Lin^–^ cells isolated from bleomycin-instilled Col1-CreERT2;Fas^–/–^ and Col1-CreERT2;Fas^+/+^ mice at 3 and 6 weeks. We also sorted Lin^–^ cells from naive Col1-CreERT2;Fas^fl/fl^ mice for comparison. Bulk RNA-seq showed that compared, with naive mice, bleomycin-instilled mice of both genotypes showed an expected increase in profibrotic gene expression and fibrosis-associated pathways at 3 weeks (data not shown). Principal Component Analysis (PCA) of highly variable genes partitioned samples by time of harvest after bleomycin instillation along the PCA-1 (30% variance) and by genotypic Fas deficiency along the PCA-4 (5% variance) (Figure 8A). There were no significant differences in profibrotic gene signatures in Lin^–^ cells isolated from bleomycin-instilled Col1-CreERT2;Fas^–/–^ and Col1-CreERT2;Fas^+/+^ mice at 3 weeks. Pathway analysis of 393 upregulated differentially expressed genes (DEGs) at 6 weeks revealed that the most significantly enriched pathways in Col1-CreERT2;Fas^–/–^ mice were associated with ECM organization (*P* = 6.32 × 10^–13^), collagen organization (*P* = 6.59 × 10^–9^), endodermal cell differentiation (*P* = 1.45 × 10^–5^), and ECM-receptor interaction (*P* = 0.0014) when compared with Col1-CreERT2;Fas^+/+^ mice (Figure 8B, Supplemental Figure 9, and Supplemental Table 2). The transcripts that contributed to these pathways included the typical profibrotic genes Col1a1, Col5a1, Col6a1, Col7a1, Col8a1, Col11a1, Eln, and Loxl2 (Figure 8C). By contrast, Lin^–^ cells from the Col1-CreERT2;Fas^+/+^ mice, which were undergoing homeostatic fibrosis resolution at 6 weeks, showed enrichment for regulation of cell migration (*P* = 0.0079) and included genes involved in wound healing (Epb41l4b, Erbb3, Itgb3, Wnt7A, and Nrg1) and Wnt signaling (Wnt10B, Fzd5, Wnt3A, Wnt7A, Sost, Rspo4, and Wnt4) (Figure 8B, Supplemental Figure 9, and Supplemental Table 2).

**Figure 9:**
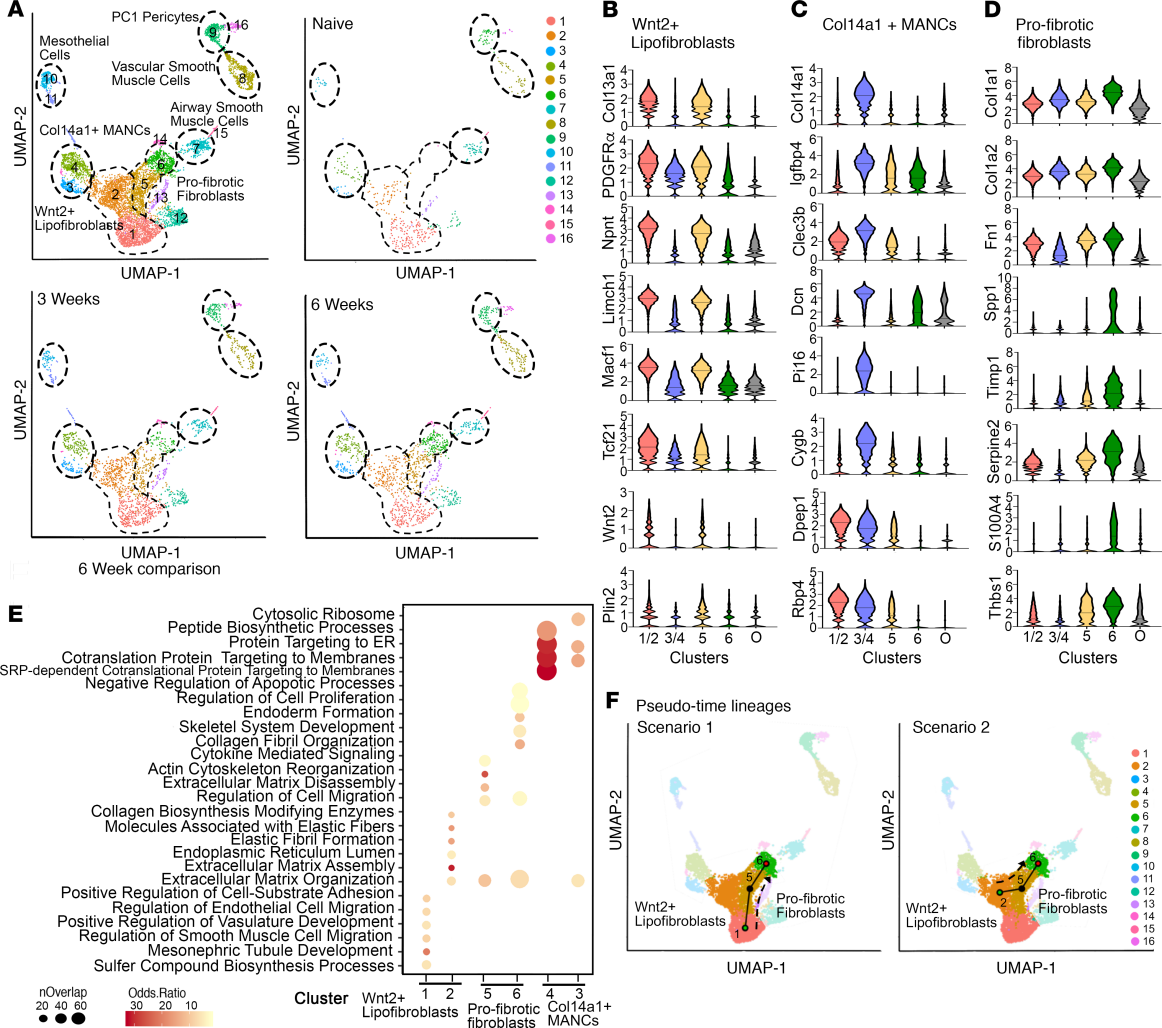
scRNA-seq reveals distinct fibroblast populations in naive and fibrotic lungs. (**A**) UMAP plot of all samples and the identified population clusters and from Fas-sufficient naive mice 3 and 6 weeks after bleomycin. (**B**) Violin plots showing genes highly associated with Wnt2^+^ Lipofibroblasts (clusters 1 and 2). (**C**) Violin plots showing genes highly associated with Col14a1^+^ mesenchymal alveolar niche cells (MANCs) (clusters 3 and 4). (**D**) Violin plots showing genes highly associated with profibrotic fibroblasts (clusters 5 and 6). O, combined expression of other clusters (clusters 3, 6, 8, 9, 11, 13, 15). (**E**) Representation of enrichment for GO pathway categories for clusters 1, 2, 3, 4, 5, and 6, a total of 6 weeks after bleomycin. (**F**) Pseudotime trajectory analysis for 2 scenarios. Violin plots show median. *n* = 4 pooled mice/group.

To investigate heterogeneity among lung fibroblast populations, we conducted scRNA-seq on sorted Lin^–^ cells from naive mice and bleomycin-instilled Col1-CreERT2;Fas^–/–^ and Col1-CreERT2;Fas^+/+^ mice at 3 and 6 weeks (Figure 9A). Although our scRNA-seq was conducted on purified Lin^–^ fibroblasts, minor clusters of microvascular and lymphatic endothelial cells, alveolar epithelial cells, hematopoietic cells, and erythroid cells were also identified (Supplemental Figure 10, A and B). To focus our analysis on fibroblast populations, we removed these nonfibroblastic cell clusters, resulting in a final data set of cell populations highly expressing canonical fibroblast genes (PDGFRα, PDGFRβ, and Acta2; Figure 9A and Supplemental Figure 10C). With this strategy, we identified 7 major cell groupings comprising 11 cell clusters in naive mice and fibrotic mice (Figure 9A and Supplemental Figures 11 and 12).

Clusters 1 and 2 bore similarity to previously described Col13a1-expressing and Wnt2^+^ fibroblasts (29, 30). These clusters also showed enriched expression of typical lipofibroblast genes, including Plin2, Tcf21, and PDGFRα, as well as other genes, including Limch1 and Npnt (Figure 9B and Supplemental Figure 11). We collectively define clusters 1 and 2 as a single population of Wnt2^+^ lipofibroblasts. Clusters 3 and 4 displayed similarity to both Col14a1-expressing fibroblasts and Axin2^+^PDGFRα^+^ mesenchymal alveolar niche cells (MANCs) (29, 30) and were defined by enriched expression of Col14a1, Dcn, Igfbp4, Macf1, Cygb, Clec3b, and Rbp4 (Figure 9C and Supplemental Figure 11). Thus, we define clusters 3 and 4 as a single population of Col14a1^+^ MANCs. Cluster 7 cells were identified as airway smooth muscle cells and/or PC2 pericyte–like cells based on enriched expression of Hhip, Must1, Aspn, Enpp2, Lum, and Acta2 (Figure 9A and Supplemental Figure 12D), while cluster 8 cells were identified as vascular smooth muscle cells and Axin^+^ myogenic progenitor cells based on enriched expression of Actc1, Acta2, Myh11, CNN1, Itih4, Lmod1, and Lgr6 (Figure 9A and Supplemental Figure 12B). Cluster 9 cells were identified as PC1 pericyte–like cells, which displayed abundant expression of Pdzd2, Cox4i2, Adcy8, and Higd1b, along with the highest expression of PDGFRβ among all clusters (Supplemental Figures 11B and 12C) (29, 45, 46); clusters 10 and 11 cells were identified as a single group of mesothelial cells based on enriched expression of Upk3b, Lrrn4, Msln, Gpm6a, and Rspo1 (Figure 9A and Supplemental Figure 12E). An additional, potentially novel population comprising clusters 5 and 6 was identified in the lungs of bleomycin-instilled mice (Figure 9A). Cluster 5 exhibited increased expression of archetypal profibrotic genes, including Col1a1, Col1a2, and Tbsp1, whereas cluster 6 was defined by enrichment with additional and distinct profibrotic genes, including Spp1, Fn1, Cthrc1, and Thbs1. In addition, cluster 6 exhibited the highest expression of PDGFRα, as well as substantially increased Acta2 expression among fibroblast clusters (Figure 9D). Based on these characteristics, we define clusters 5 and 6 as profibrotic fibroblasts.

Next, we compared the gene expression patterns among the Wnt2^+^ lipofibroblasts, Col14a1^+^ MANCs, and profibrotic fibroblast subsets in bleomycin-instilled Col1-CreERT2;Fas^–/–^ and Col1-CreERT2;Fas^+/+^ mice at 3 and 6 weeks. To verify efficient deletion of Fas in Col1-CreERT2;Fas^–/–^ mice, we compared the gene expression pattern of the deleted exon 9 between Col1-CreERT2;Fas^–/–^ and Col1-CreERT2;Fas^+/+^ mice at 3 and 6 weeks. Expression of exon 9 was significantly reduced (*P* < 0.001), and the number of cells expressing exon 9 was decreased 4.8-fold (from 39% to 8%) at 3 weeks and 2.3-fold (from 43% to 19%) at 6 weeks in Fas-deficient cells (Supplemental Figure 13). Exon 9 was deleted similarly in all clusters in Col1-CreERT2;Fas^–/–^ mice (data not shown). Similar to the differential expression analysis seen by bulk RNA-seq, Fas deficiency in fibroblasts had little impact on the induction of genes related to profibrotic pathways at 3 weeks in any of the fibroblast subsets, consistent with our data showing that fibroblast Fas deficiency had no effect on the development of fibrosis. By contrast, comparison of specific fibroblast populations between Col1-CreERT2;Fas^–/–^ and Col1-CreERT2;Fas^+/+^ mice at 6 weeks revealed fibrosis-associated DEGs in the profibrotic fibroblasts (clusters 5 and 6; 40 genes) and the Wnt2^+^ lipofibroblasts (clusters 1 and 2; 95 genes), but not in the Col14a1^+^ MANCs (clusters 3 and 4). Within the profibrotic fibroblasts (clusters 5 and 6) of Col1-CreERT2;Fas^–/–^ mice at 6 weeks, gene ontology pathways associated with ECM organization (*P* = 1.916 × 10^–29^), regulation of cell migration and actin cytoskeleton reorganization (*P* = 5.711 × 10^–7^), and negative regulation of apoptotic processes (*P* = 3.599 × 10^–6^) were also highly enriched, indicating that profibrotic signatures persisted in this Fas-deficient profibrotic subset (Figure 9E and Supplemental Table 3). Additionally, the upregulated DEGs from the Wnt2^+^ lipofibroblasts were enriched for gene ontology pathways associated with elastic fibril formation (*P* = 0.0072) and collagen biosynthesis modifying enzymes (*P* = 0.0335), and containing other ECM assembly genes (Col28A1, Adamts2, Col13A1, Col4A5, Cst3, Rcn1, Sparcl1, Igfbp7, Gas6, Vcam1, Mmp2, Mmp3, Loxl1, and Fbln5). Gene ontology pathways associated with peptide biosynthesis processes, and targeting of proteins to the ER and membranes, were enriched in Col14a1^+^ MANCs fibroblasts (Figure 9E and Supplemental Table 3). Thus, both the profibrotic fibroblasts and the Wnt2^+^ lipofibroblasts retained profibrotic gene expression patterns in the absence of Fas signaling.

Lastly, to provide insight into the potential progenitors and lineages leading to the profibrotic fibroblast populations (clusters 5 and 6) that emerge with injury and fibrosis, we performed in silico trajectory analyses of our single cell data. We included the Wnt2^+^ lipofibroblast population (clusters 1 and 2) in this analysis as potential progenitors, given their related expression profile, exemplified by the connected nature of clusters 1, 2, 5, and 6 in Uniform Manifold Approximation and Projection (UMAP) space. Further supporting cluster 1 as a progenitor for clusters 5 and 6, we found the frequency of this cluster decreased from 30% in naive animals to 20% and 14% as fibrosis developed at 3 and 6 weeks, respectively. Pseudotime trajectory analysis inferred 2 major trajectories: Lineage 1, proceeding from cluster 1 through cluster 5 to cluster 6, and Lineage 2, proceeding from cluster 2 through cluster 5 to cluster 6 (Figure 9F). Through clustering of pseudotime-associated genes, we computed the trajectories into early, mid, and late phases. The pseudotime-associated genes for the early and mid phases of Lineage 1 exhibited enrichment for profibrotic pathways, including lung fibrosis (*P* = 0.036), ECM-receptor interactions (*P* = 8.18 × 10^–8^), focal adhesion formation (*P* = 3.4 × 10^–6^), TGF-β signaling pathways (*P* = 0.05327), and matrix metalloproteinases (*P* = 0.01727) (Figure 9F, Supplemental Figure 14, and Supplemental Table 4). Similar pathways were enriched during the early and mid phases of the Lineage 2, but also included genes involved in glutathione metabolism (Gpx3) and VEGF signaling (Hspb1). Late trajectory genes included matrix metalloproteinase genes (Mmp3, Timp3) and genes involved in IGF1 signaling (Igfbp3, Igfbp6) (Figure 9F, Supplemental Figure 14, and Supplemental Table 5). Thus, pseudotime analysis suggests that the profibrotic fibroblasts (clusters 5 and 6) develop from the Wnt^+^ lipofibroblasts (clusters 1 and 2) in naive lungs. Taking into consideration both the bulk and scRNA-seq data, our findings suggest that once each fibroblast cluster has undergone its unique profibrotic programming response, the profibrotic gene expression profile remains largely unchanged as long as fibrosis persists.

## Discussion

Substantial progress has been made in understanding the mechanisms of alveolar epithelial injury and its role in driving fibroblast recruitment, proliferation, and excessive ECM deposition in the distal lung. However, the mechanisms that distinguish profibrotic fibroblast persistence and ECM accumulation in the context of progressive pulmonary fibrosis seen in IPF from the fibrosis resolution and lung regeneration that occurs in the setting of recovery from ARDS remain unclear. Here, we show that Fas deletion in fibroblasts inhibits fibroblast apoptosis, impedes homeostatic fibrosis resolution, maintains profibrotic transcriptomic fibroblast gene expression programing, and permits fibroblast persistence and enduring pulmonary fibrosis. Taken together, these findings suggest that Fas signaling plays a fundamentally important, physiologic role in the elimination of profibrotic lung fibroblasts during fibrosis resolution in vivo. Furthermore, our results suggest that impaired Fas signaling through Fas downregulation, or by increased expression of antiapoptotic proteins that inhibit proapoptotic Fas signaling (11, 17–20), may lead to critical deviations in lung fibrosis outcome from resolution to persistence.

Two approaches were used to delete Fas in fibroblasts. Dermo1 is developmentally expressed in lung mesenchyme (26). Thus, fibroblasts in Dermo1-Cre;Fas^–/–^ mice are Fas deficient at birth (47–49). We also conditionally deleted Fas in fibroblasts during bleomycin-induced fibrosis development in Col1-CreERT2;Fas^–/–^ mice. With both approaches, bleomycin robustly induced pulmonary fibrosis between 2 and 4 weeks. However, whereas Fas-sufficient mice underwent homeostatic fibrosis resolution by 6–9 weeks (9, 10, 24, 50), fibroblast Fas deficiency in both Dermo1-Cre;Fas^–/–^ and Col1-CreERT2;Fas^–/–^ mice inhibited homeostatic fibrosis resolution and led to persistent pulmonary fibrosis for at least 9 weeks.

Fibroblast Fas deficiency profoundly inhibited both the apoptosis and elimination of lung fibroblasts during homeostatic fibrosis resolution. Using a potentially novel flow cytometry strategy for pulmonary fibroblasts, based on previous studies describing CD90^+^, CD90^–^, and CD26^+^ fibroblasts in lung and skin (35, 51), we found that, among Fas-deficient lung fibroblasts, CD26^+^ and CD90^–^CD26^–^ fibroblasts remained the most persistent and elevated subsets at 6 and 9 weeks. CD90^+^ fibroblasts underwent a modest early expansion but remained significantly elevated at 6 and 9 weeks in the absence of Fas signaling. Furthermore, whereas apoptotic S100A4^+^ and α-SMA^+^ fibroblasts were detected in the lungs of bleomycin-instilled Dermo1-Cre;Fas^+/+^ and Col1-CreERT2;Fas^+/+^ mice at 3 and 4.5 weeks, they were not detected in Dermo1-Cre;Fas^–/–^ and Col1-CreERT2;Fas^–/–^ mice, suggesting that Fas signaling plays an essential role in fibroblast apoptosis during homeostatic fibrosis resolution. The lungs of naive Dermo1-Cre;Fas^–/–^ mice were found to have basally elevated numbers of Lin^–^PDGFRα^+^ cells compared with naive Dermo1-Cre;Fas^+/+^ mice, with the majority being CD26^+^. These findings suggest that Fas signaling may be of greater relevance to basal turnover of CD26^+^ fibroblasts compared with CD90^+^ and CD90^–^CD26^–^ fibroblasts. However, we did not observe spontaneous lung or skin fibrosis in these mice or in aged (i.e., >1-year-old) Dermo1-Cre;Fas^–/–^ mice (data not shown). We also noted that fibroblast Fas deficiency in Dermo1-Cre;Fas^–/–^ and Col1-CreERT2;Fas^–/–^ mice had no effect on the initial bleomycin-induced fibroblast expansion at 1.5 and 3 weeks. Previous in vitro studies have suggested that Fas signaling during this period contributes to profibrotic fibroblast proliferation via cFLIP_L_– and TRAF2-induced NF-κB activation (20). Our use of genetic in vivo approaches to delete Fas in fibroblasts challenges this notion. Taken together, our results suggest that Fas signaling plays a necessary role in fibroblast apoptosis and elimination during homeostatic fibrosis resolution following bleomycin-induced lung injury and likely contributes to fibroblast turnover in naive mice.

Much remains to be understood about the functions of the increasingly heterogeneous lung fibroblast subsets. We initially studied how Fas signaling affects CD90^+^, CD26^+^, and CD90^–^CD26^–^ fibroblasts. CD90 is expressed by Tcf21-expressing lipofibroblasts and Col13a1 interstitial matrix–producing fibroblasts (52). In the context of bleomycin-induced pulmonary fibrosis, CD90-deficient mice phenocopy the impaired homeostatic fibrosis resolution described herein, and lung fibroblasts from CD90-deficient mice are resistant to Fas-induced apoptosis (10, 53). CD26 has been reported to be expressed on dermal fibroblasts that synthesize and deposit ECM during scar formation (35), and we have shown it to identify a CD90^–^ fibroblast subset. We have also shown that CD90^+^, CD26^+^, and CD90^–^CD26^–^ fibroblasts from bleomycin-instilled mice exhibit increased Col1a1 promoter activity, peaking at 3 weeks in Col1-GFP reporter mice. However, whereas GFP expression had returned to baseline level by 6 weeks in Fas-sufficient fibroblasts from Col1-CreERT2;Fas^+/+^;Col1-GFP mice, GFP expression remained elevated in Fas-deficient CD90^+^, CD26^+^, and CD90^–^CD26^–^ fibroblasts in Col1-CreERT2;Fas^–/–^;Col1-GFP mice. Similarly, α-SMA promoter activity remained elevated in CD90^+^ fibroblasts in bleomycin-instilled Fas-deficient Col1-CreERT2;Fas^–/–^;α-SMA–RFP reporter mice at 6 weeks compared with CD90^+^ fibroblasts from Fas-sufficient Col1-CreERT2;Fas^+/+^;α-SMA–RFP reporter mice. Thus, loss of Fas signaling in fibroblasts not only prevents fibroblast apoptosis, but it prolongs functional gene expression from profibrotic Col1a1 and α-SMA promoters in surviving cells.

Bulk and scRNA-seq provided further insight into the transcriptional profiles and, by inference, the functions of the Fas-deficient fibroblasts that remained in persistently fibrotic lungs. Analysis of bulk RNA-seq data from Fas-sufficient and Fas-deficient fibroblasts showed marked similarities in profibrotic gene expression and pathway enrichment at 3 weeks compared with naive mice. scRNA-seq provided additional insight. We identified 2 major fibroblast subsets in the lungs of naive mice. Wnt2^+^ lipofibroblasts (clusters 1 and 2) displayed similarity to Wnt2^+^ (30) and Col13a1 fibroblasts (29), but they were also enriched with transcripts encoding lipofibroblast genes including Plin2 (29, 52). Col14a1^+^ MANCs were similar to Axin^+^PDGFRα^+^ and Col14a1^+^ fibroblasts (29, 30). At the peak of fibrosis, Wnt2^+^ lipofibroblasts from both Fas-sufficient and Fas-deficient mice displayed increased expression of typical profibrotic genes. In contrast, gene expression profiles in Col14a1^+^ MANCs from bleomycin-instilled mice did not display a gene expression profile associated with the development or maintenance of fibrosis, and very few DEGs were found in comparison with lung fibroblasts from naive mice, as previously reported (30). We also identified a third, potentially novel, profibrotic fibroblast population (clusters 5 and 6) that was present in the lungs of both Fas-sufficient and Fas-deficient bleomycin-instilled mice but was absent from the lungs of naive mice. Our data support the conclusion that these populations arise from the Wn2t^+^ lipofibroblasts (clusters 1 and 2) present in naive lungs. In silico trajectory analysis provided 2 possible differentiation paths, initiating with either cluster 1 or 2, and passing through cluster 5 to generate the profibrotic cluster 6 cells.

Analysis of the lung gene expression profiles in fibroblasts isolated from bleomycin-instilled Col1-CreERT2;Fas^+/+^ mice at 6 weeks showed enrichment in pathways associated with regulation of cell migration and motility, wound healing, Wnt signaling, and epithelial cell development, consistent with homeostatic fibrosis resolution. However, the Wnt2^+^ lipofibroblasts (clusters 1 and 2) and the profibrotic fibroblasts (clusters 5 and 6) isolated from fibrotic bleomycin–instilled Col1-CreERT2;Fas^–/–^ mice continued to express profibrotic gene expression patterns and pathways at 6 weeks, indicating that their profibrotic programming had been preserved in the absence of Fas signaling.

Studies into the role of impaired Fas signaling in tissue injury and fibrosis began to appear in the 1990s following the identification of a spontaneous inactivating Fas mutation in *lpr* mice (54). These mice provided a new tool to address the consequences of Fas inactivation in vivo. However, they led to conflicting results when used to investigate the role of Fas in bleomycin-induced acute lung injury and fibrosis (55, 56). Loss of Fas signaling in *lpr* mice was also reported to reduce hepatocyte apoptosis and subsequent hepatic fibrosis after bile duct ligation (57). A clear limitation of these early studies is that whole-body Fas inactivation does not allow for adequate modeling of the temporally distinct complex cellular interactions that are required to promote injury, fibrosis, and repair where the impact of loss of Fas signaling in alveolar epithelial cells may be quite different from that in fibroblasts or immune cells. The use of lineage-restricted and conditional Fas inactivation in fibroblasts, as applied herein, circumvents this problem.

In summary, our data suggest that, following initial expansion and profibrotic programming of fibroblasts during the development of fibrosis, fibroblasts deficient in Fas maintain their profibrotic transcriptomic programming in their persistent state. Therefore, autonomous Fas-induced apoptosis and clearance of profibrotic fibroblasts during fibrosis resolution play necessary roles in homeostatic fibrosis resolution, whereas impaired Fas signaling is sufficient to maintain both their presence in persistently fibrotic lungs and their profibrotic programming. We speculate that loss of the ability of fibroblasts to undergo Fas-induced apoptosis represents a decisive checkpoint at which the beneficial, homeostatic resolving fibrotic response might be diverted to the persistent and potentially more harmful progressive fibrosis seen in IPF patients.

## Methods

### Mouse strains.

To enact lineage-specific deletion of Fas, we bred mice that expressed endogenous Cre recombinase driven by the *Dermo1* (*Twist2*) promoter (008712 - B6.129X1-*Twist2^tm1.1(cre)Dor^*/J; The Jackson Laboratory) or a tamoxifen-inducible Cre recombinase driven by the Col1a1 promoter (016241 - B6.Cg-Tg[Col1a1-cre/ERT2]1Crm/J; The Jackson Laboratory) to mice with floxed Fas alleles (exon 9) (007895- C57BL/6-*Fas^tm1Cgn^*/J; The Jackson Laboratory). The final genotypes of the mice were *Dermo1-Cre;Fas^fl/fl^* and *Col1a1-Cre-ERT2;Fas^fl/fl^*, respectively. To track real-time expression of Col1a1 and α-SMA, we used Col1a1-GFP and α-SMA–RFP mice (gifts from David Brenner, University of California San Diego, San Diego, California, USA; ref. 40). The Col1a1-GFP and α-SMA–RFP mice were bred to *Col1a1-Cre-ERT2;Fas^fl/fl^* to allow for Fas deletion and florescence expression of Col1a1 or α-SMA. For all mice bred to the Col1a1-ERT2 promoter, either corn oil or tamoxifen (0.25 mg/g body weight in corn oil, MilliporeSigma) was given by i.p. injection starting at day 4 after bleomycin. Mice received 6 injections between days 4 and 21 (Figure 2A). Deletion of Fas after Cre recombination following tamoxifen was confirmed by PCR (Supplemental Figure 2; Cre forward, 5′ - GAGTGAACGAACCTGGTCGAAATCAGTGCG - 3′; Cre reverse, 5′ - GCATTACCGGTCGATGCAACGAGTGATGAG - 3′; Fas Del forward, 5′ - GTCCTCTATTATCCTCATCATGAG - 3′; Fas Del reverse, 5′ - GGCTTTGGAAAGGAATTTCCTCCTAAGAGG - 3′; Fas LoxP forward, 5′ - CCTTCCATTGATGGACAGTTC - 3′; Fas LoxP reverse, 5′ - TTAAAAGGCTTTGGAAAGGAA - 3′) (Integrated DNA Technologies).

### Primary fibroblasts.

Primary lung fibroblasts were isolated from healthy murine lungs as previously described (17, 58). They were maintained in 10% DMEM, grown on plastic, and used in experiments between passages 3 and 8.

### Assessment of fibrotic lung disease.

Pulmonary fibrosis was initiated by the intratracheal instillation of 50 μL of bleomycin (1.5 U/kg, Amneal Biosciences) to anesthetized mice, as previously described (24). Fibrosis was assessed by lung measurements of collagen in the upper right lobe (hydroxyproline). Briefly, lungs were homogenized in PBS and hydrolyzed overnight at 120°C using 12M HCl. Assessment of hydroxyproline was determined by the absorbance at 500 nm on a microplate reader (Epoch2 BioTek Instruments). Histology was evaluated by H&E staining and (PSR of sections from the left lung as previously described (24). Images were taken on an upright Olympus BX51.

### Flow cytometry and cell sorting.

Single cell suspensions were obtained from perfused, enzymatically dispersed lungs. Briefly, a digestion mixture of collagenase (450 U/mL, MilliporeSigma), Dispase (5 U/mL, Worthington Biochemical), and Elastase (4 U/mL, Worthington Biochemical) was instilled into the right lung and incubated at 37°C for 25 minutes. Lungs were chopped, and tissue pieces were washed in 10% FBS containing DMEM, followed by a secondary digestion in 0.1% Trypsin-EDTA with 0.33 U/mL DNaseI (Worthington Biochemical) for 20 minutes at 37°C. Dissociated tissue was washed in DMEM containing 10% FBS, and single cell suspensions were filtered prior to staining (37). Cells were stained with fluorescently tagged monoclonal antibodies against CD45 (catalog 17-0451-82), CD326/EpCAM (catalog 17-5791-80), CD90 (catalog 48-0902-80), CD26 (catalog 45-0261-80), PDGFR/CD140a (catalog 25-1401-82) (all from Thermo Fisher Scientific), and CD31 (catalog 541814, BD Biosciences) at a 1:200 dilution. Cell analysis data were acquired with the LSRFortessa (BD Biosciences) and analyzed with FlowJo 2 software (Tree Star Inc.).

Prior to cell sorting, single cell suspensions were enriched for Lin^–^ cells by incubating with CD45 (catalog 130-052-301), CD31 (catalog 130-097-418), and CD326 (catalog 130-105-958) MicroBeads and purified off of LS Column per manufacturer instructions (Miltenyi Biotec). Single cells were sorted on the FACSAria Fusion (BD Biosciences) as doublet excluded, DAPI^–^, CD45^–^, CD31^–^, and CD326^–^ prior to single cell and bulk RNA-seq of the Lin^–^ population.

### Immunofluorescence staining.

Formalin-fixed, paraffin-embedded sections were deparaffinized and rehydrated followed by antigen retrieval using citrate buffer. Nonspecific binding was reduced by incubation in 10% blocking serum and mouse IgG serum (Vector Laboratories). Primary anti–rabbit S100-A4/FSP1 antibody (07-2274, MilliporeSigma) was used at a 1:500 dilution, and primary anti–mouse α-SMA antibody (clone 1A4, MilliporeSigma) was used at a 1:1000 dilution overnight at 4°C, followed by incubation with fluorescently tagged goat anti–mouse A647 (catalog A21236) and donkey anti–rabbit A555 (catalog A31572) secondary antibodies at 1:100 dilution (Invitrogen). TUNEL staining to detect apoptotic cells was completed prior to antibody staining, per manufacturer instructions (Promega). TUNEL^+^ cells were counted and averaged from 10 images from each animal. Images were captured in fibrotic areas using FSP1^+^ staining as an indicator of fibroblast accumulation. TUNEL^+^ cells located in the airway epithelium were excluded from the analysis. Frozen 4% PFA-fixed OCT (Thermo Fisher Scientific) sections were mounted with Fluoroshield Mounting Media containing DAPI (Vector Laboratories). Images were acquired on a Zeiss Axioplan 2 epi-fluorescence microscope and analyzed with Axiovision software (Zeiss).

### Bulk RNA-seq.

Lin^–^ cells were obtained by FACS sorting. At least 300,000 cells were collected per population. Purified cells were pelleted and lysed, and RNA was precipitated using TRIzol (Thermo Fisher Scientific), followed by purification of the aqueous layer using a RNeasy Micro Kit (Qiagen Sciences). A modified Kapa Biosystems (Wilmington) KAPA Stranded mRNA-Seq kit for whole transcriptome libraries was used to primarily target all polyA RNA. After mRNA (poly-A) isolation, first- and second-strand cDNA synthesis, adaptor ligation, amplification, and bead templating occurred. Once validated, the libraries were sequenced as barcoded pooled samples and processed for next-generation sequencing (NovaSeq 6000; Illumina platform). The data set has been deposited in the National Center for Biotechnology Information/Gene Expression Omnibus under accession no. GSE161648 (https://www.ncbi.nlm.nih.gov/geo/query/acc.cgi?acc=GSE161648).

### scRNA-seq.

All single cell capture and library preparation was performed at the University of Colorado Cancer Center Microarray and Genomics Core. Cells were loaded onto a 10× Genomics microfluidics chip and encapsulated with barcoded oligo-dT–containing gel beads using the 10× Genomics Chromium controller and single cell libraries constructed per the manufacturer’s instructions. Samples were sequenced on a NovaSeq 6000 Illumina platform instrument. An average of 2741 cells per sample were analyzed with an average postnormalized mean read/cell of 312,995. The data set has been deposited in the National Center for Biotechnology Information/Gene Expression Omnibus under accession no. GSE161648 (https://www.ncbi.nlm.nih.gov/geo/query/acc.cgi?acc=GSE161648).

### Bulk RNA-seq analysis.

To improve downstream mapping quality, raw sequencing reads were trimmed using skewer with parameters (end quality [3′ end quality trimming] = 15, mean quality [reads filtered by average quality] = 25, minimum quality [specifies lowest mean quality value allowed before trimming] = 30). Trimmed reads were aligned to the mouse reference genome GRCm38 using Hisat2. Gene quantification was performed with htseq-count using GRCm38 ensembl v84 GTF. Differential expression analysis between groups was conducted with R package DESeq2. Pathway analysis was conducted with Enricher (59, 60).

### Single cell clustering and trajectory analysis.

Initial processing of 10× scRNA-seq data — including cell demultiplexing, alignment to the mouse genome GRCm38, and UMI-based quantification — was performed with Cell Ranger (version 3.0). To ensure that high-quality cells were used for downstream analysis, we removed cells with fewer than 200 genes detected or cells with greater than 20% mitochondrial reads (Supplemental Figure 15). Additionally, to remove possible doublets, we remove cells with higher than 7500 genes detected. For gene filtering, we removed lowly expressed genes (detected in fewer than 4 cells). Using the above filtering, our data set consisted of 12,043 cells and 37,887 genes. After initial clustering and visualization, 4215 cells were removed that we characterized as nonfibroblasts (Supplemental Figure 10), which left us with 7828 mesenchymal cells for downstream analysis.

Prior to clustering, we performed normalization using sctransform and integration of data sets from 5 samples (naive *Col1a1-Cre-ERT2;Fas^fl/fl^*, Col1-CreERT2;Fas^–/–^, and Col1-CreERT2;Fas^+/+^ 3 weeks after bleomycin and Col1-CreERT2;Fas^–/–^ and Col1-CreERT2;Fas^+/+^ 6 weeks after bleomycin) using a mutual nearest neighbor–based (MNN-based) approach. Clustering analysis was performed on the top 20 PCs using a shared nearest neighbor (SNN) based SLM algorithm. Visualization of the single cell expression profiles into a 2-dimensional map was computed using UMAP technique. Differential expression analysis was conducted using FindMarkers function with default options. All the analyses mentioned above is carried out with R Seurat package version 3.5.1. Pathway analysis was conducted with Enricher (59, 60).

Using the previously computed UMAP components, trajectory analysis was performed using Slingshot that builds lineages of cells that link cell clusters by fitting a minimum spanning tree (MST) onto the selected clusters, followed by the application of simultaneous principal curves to create trajectories. Clusters 1, 2, 5, and 6 were included in the trajectory analysis. Additional constraints were added by imposing cluster 1 and cluster 2 as the starting clusters and cluster 6 as the end cluster.

### Statistics.

Time course data are presented as the mean ± SEM. Data were analyzed using GraphPad Prism software Version 8 (GraphPad). Differences between conditions at specific time points were examined using Student’s unpaired 2-tailed *t* test with Welch’s correction with *P* < 0.05 considered to be significant. Box-and-whisker plots show median, minimum, and maximum values. Violin plots show median, upper, and lower quartiles. Specifics about the replicates used where *n* = individual animal replicates are available in the figure legends.

### Study approval.

All Animal studies were approved by the National Jewish Health IACUC.

## Author contributions

EFR, SC, BPB, SS, and BLE conducted the experimental work. DWHR conceived the project. EFR, MAS, and DWHR designed and planned experiments, analyzed the data, and contributed to writing the manuscript. All authors reviewed the manuscript.

## Supplementary Material

Supplemental data

## Version 1 12/08/2020

In-Press Preview

## Version 2 01/11/2021

Electronic publication
