# Supplementary material for: Loss of Fas signaling in fibroblasts impairs homeostatic fibrosis resolution and promotes persistent pulmonary fibrosis
Source: JCI Insight. 2020 Dec 8;6(1):e141618. doi: 10.1172/jci.insight.141618 (PMC7821600; doi:10.1172/jci.insight.141618)
Supplement: Supplemental data [file jciinsight-6-141618-s100.pdf]

Supplemental Figure 1

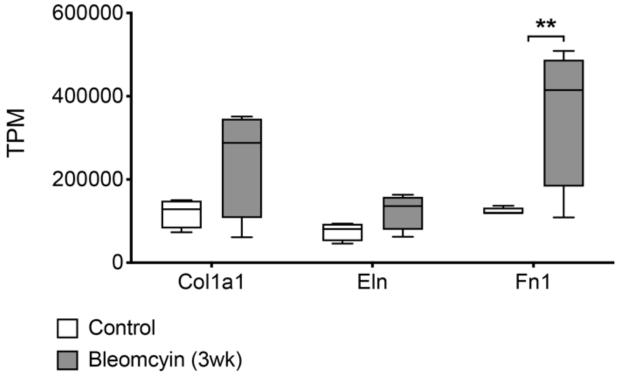

Supplemental Figure 1. Bleomycin-induced fibrosis increases pro-fibrotic gene expression in fibroblasts. Gene expression in primary fibroblasts isolated and grown in culture from lungs of control and fibrotic mice. Box-and-whisker plots show median, minimum and maximum values. n=3/group **p<0.01. 2-tailed *t* test with Welch's correction.

Supplemental Figure 2

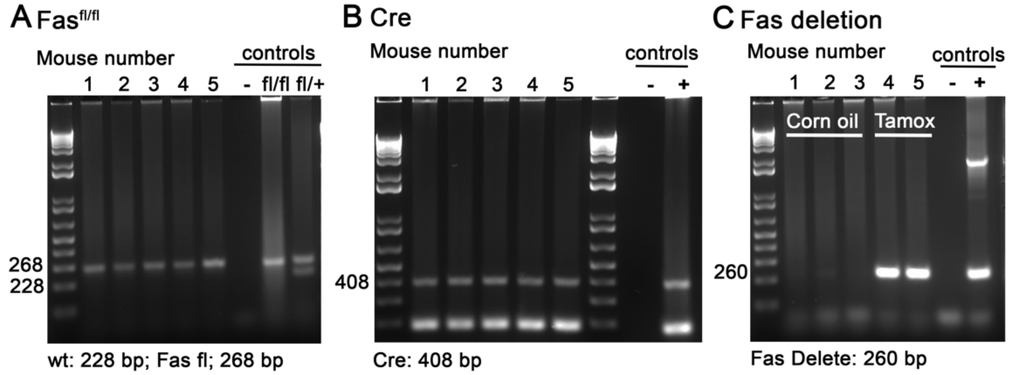

Supplemental Figure 2. Deletion of Fas under the Col1a1 promoter after tamoxifen. (A) PCR genotyping of loxp sites around exon 9 of Fas. (B) PCR genotyping of Cre. (C) PCR genotyping of Fas deletion band after *in vivo* tamoxifen treatment.

Supplemental Figure 3

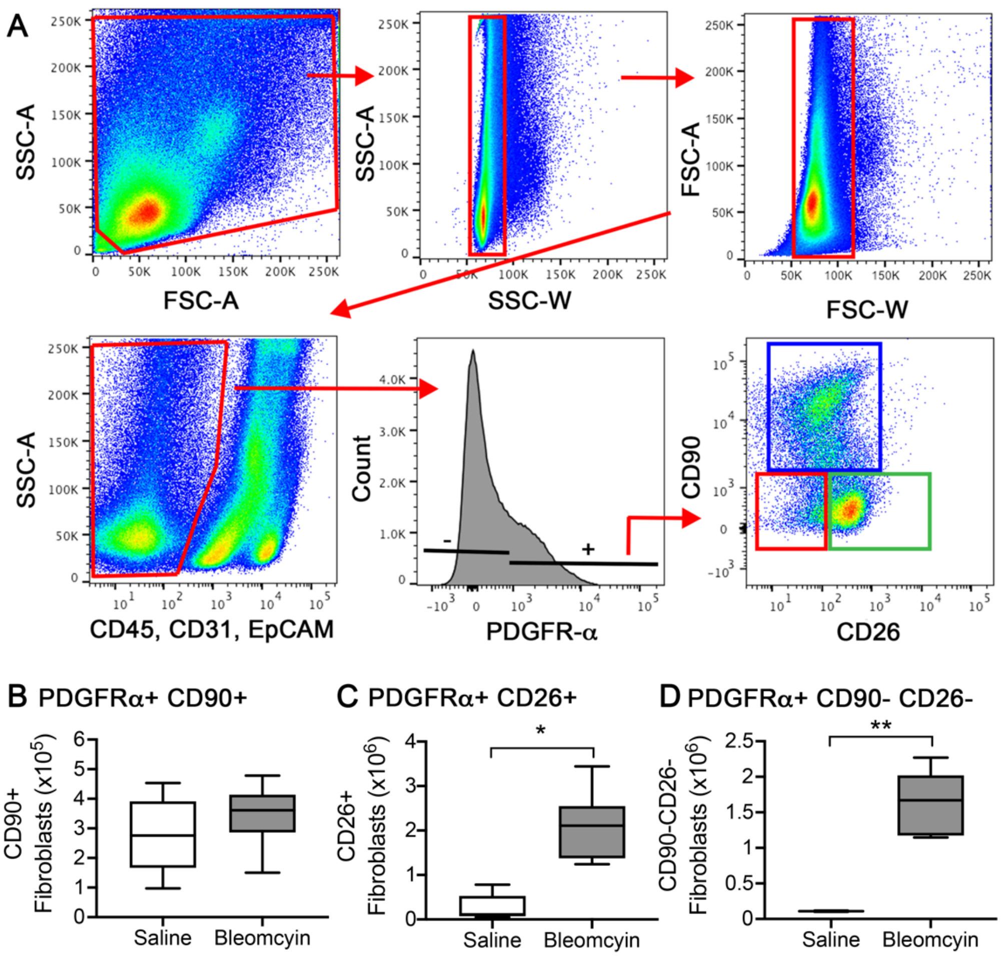

Supplemental Figure 3. Flow cytometry identification of fibroblast subpopulations in fibrotic wild type mice. (A) Flow cytometry strategy to identify fibroblast subsets. Quantitation of Lin- PDGFRα+ fibroblast sub-populations: (B) CD90+, (C) CD26+ and (D) CD90-CD26- by flow cytometry. Box-and-whisker plots show median, minimum and maximum values. n=3/group *p<0.05, **p<0.01. 2-tailed *t* test with Welch's correction.

Supplemental Figure 4

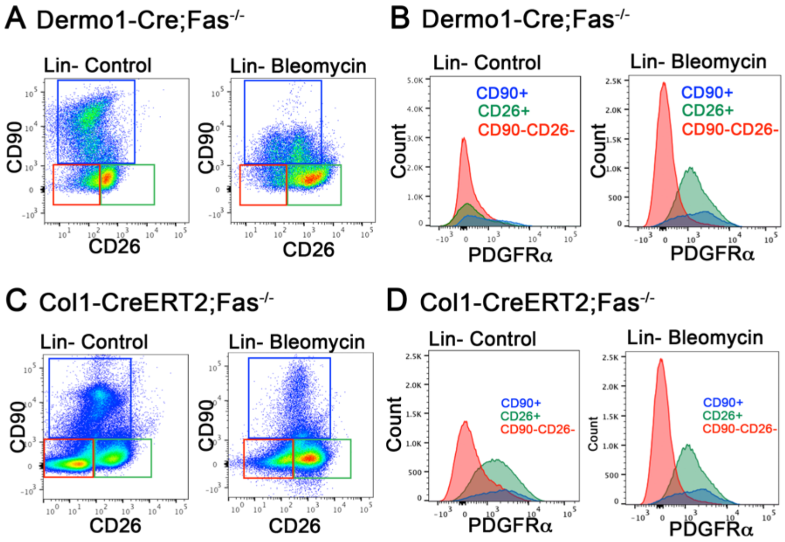

Supplemental Figure 4. Flow cytometry identification of fibroblast subpopulations in mice with Fas deletion. (A) Representative gating strategy to Lin- subsets: CD90+CD26-, CD26+CD90- and CD90-CD26- and (B) their relative expression of PDGFRα in control and after bleomycin treatment in Dermo1-Cre;Fas$^{-/-}$ mice. (C) Representative gating strategy to Lin- subsets: CD90+CD26-, CD26+CD90- and CD90-CD26- and (D) their relative expression of PDGFRα in control and after bleomycin treatment in Col1-CreERT2;Fas$^{-/-}$ mice.

Supplemental Figure 5

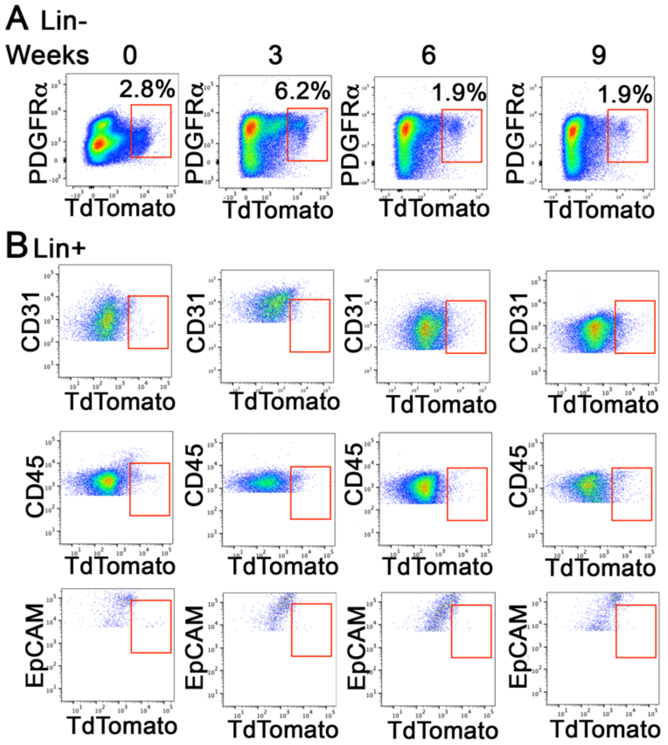

Supplemental Figure 5. Col1a1 lineage expression of TdTomato to identify collagen expressing cells. Flow cytometry analysis of Col1-CreERT2;TdTomato mice treated with tamoxifen (Figure 2A) to identify Col1 expressing cells (TdTm+) over time after bleomycin. (A) Representative flow plots of Lin(-) PDGFRα+ fibroblasts and (B) Lin+ CD31, CD45 and EpCAM cells and TdTomato expression.

Supplemental Figure 6

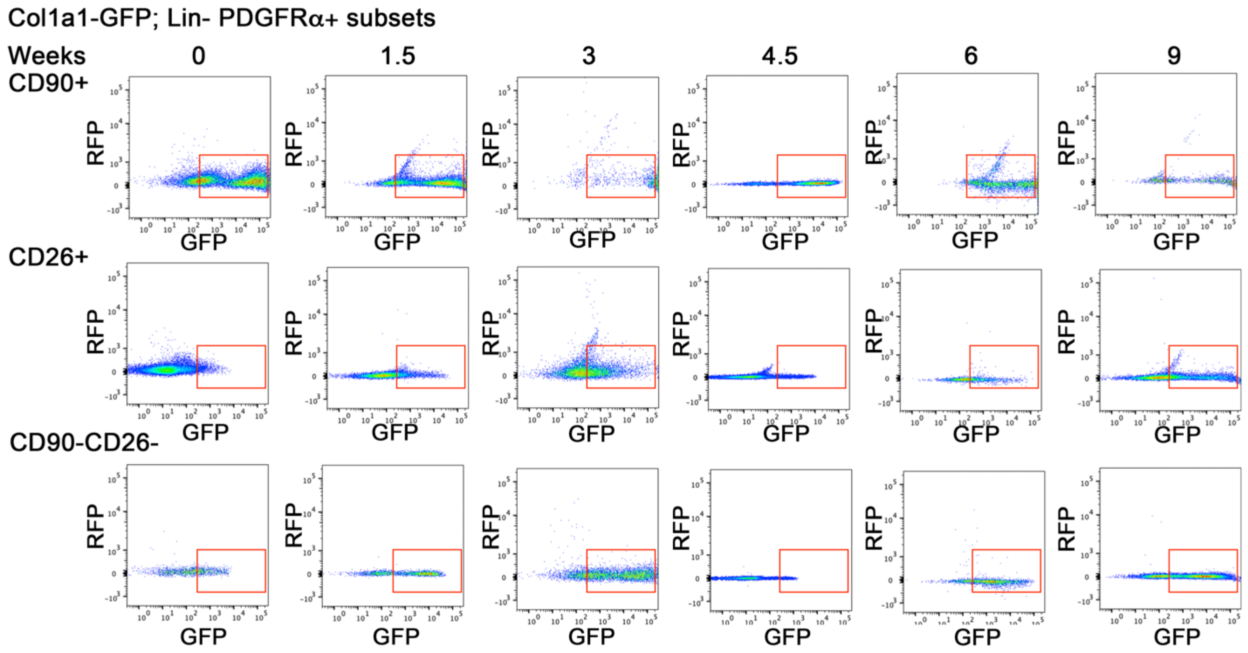

Supplemental Figure 6. Col1-GFP expression of fibroblast subsets over time after bleomycin. Representative flow cytometry plots of GFP expression in CD90+CD26-, CD26+CD90- and CD90-CD26- fibroblasts subsets in Col-GFP mice during fibrosis development and resolution.

Supplemental Figure 7

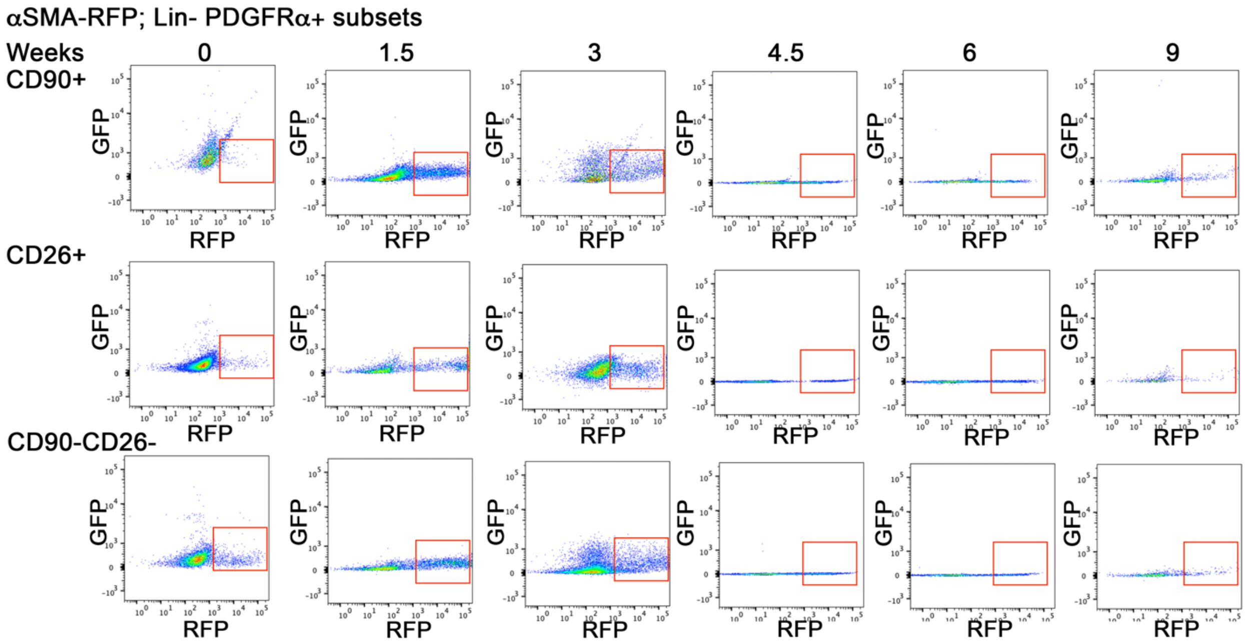

Supplemental Figure 7. αSMA-RFP expression of fibroblast subsets over time after bleomycin. Representative flow cytometry plots of RFP expression in CD90+CD26-, CD26+CD90- and CD90-CD26- fibroblasts subsets in αSMA-RFP mice during fibrosis development and resolution.

Supplemental Figure 8

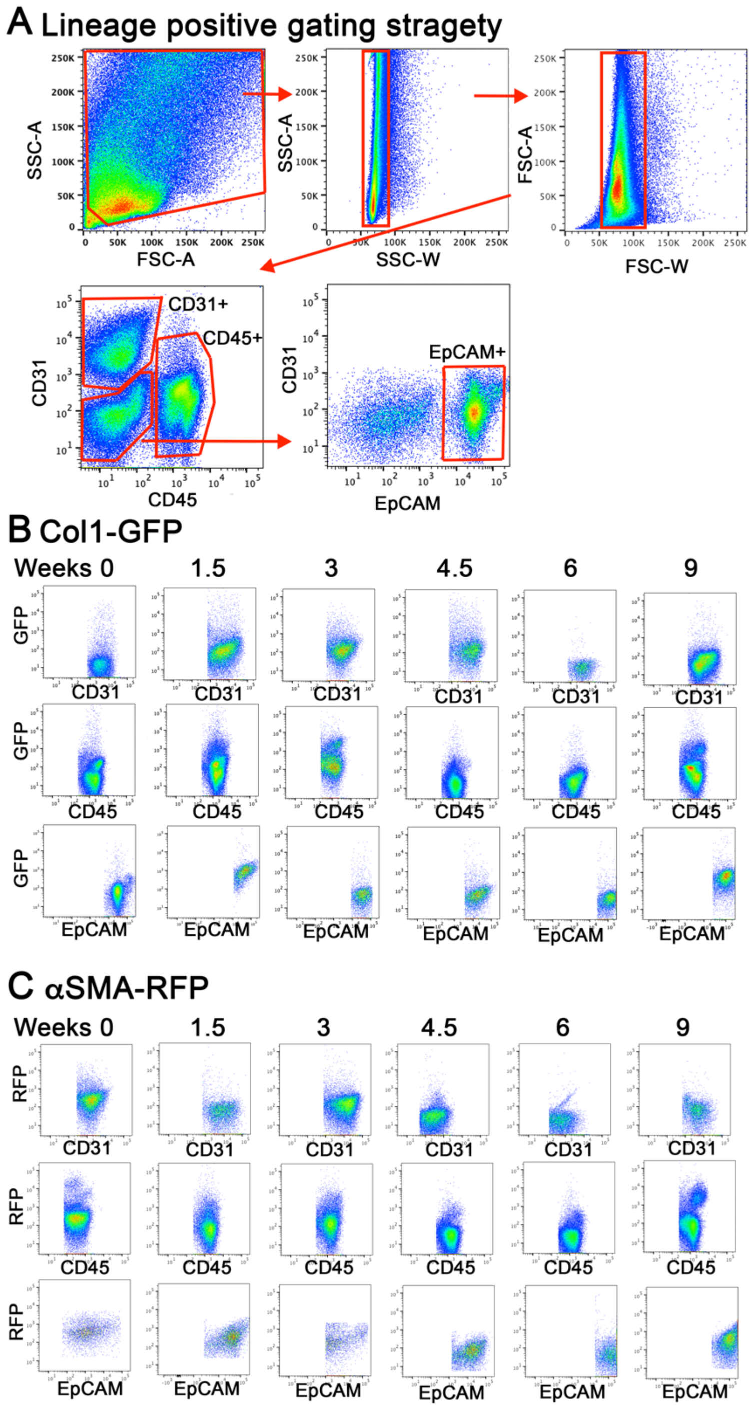

Supplemental Figure 8. Flow cytometry gating strategy for Lineage positive cells. (A) Representative gating strategy to identify lineage positive CD31, CD45 and EpCAM cells. (B) Representative flow plots of GFP expression in lineage positive cells over time after bleomycin in Col-GFP mice. (C) Representative flow plots of RFP expression in lineage positive cells over time after bleomycin in αSMA-RFP mice.

Supplemental Figure 9

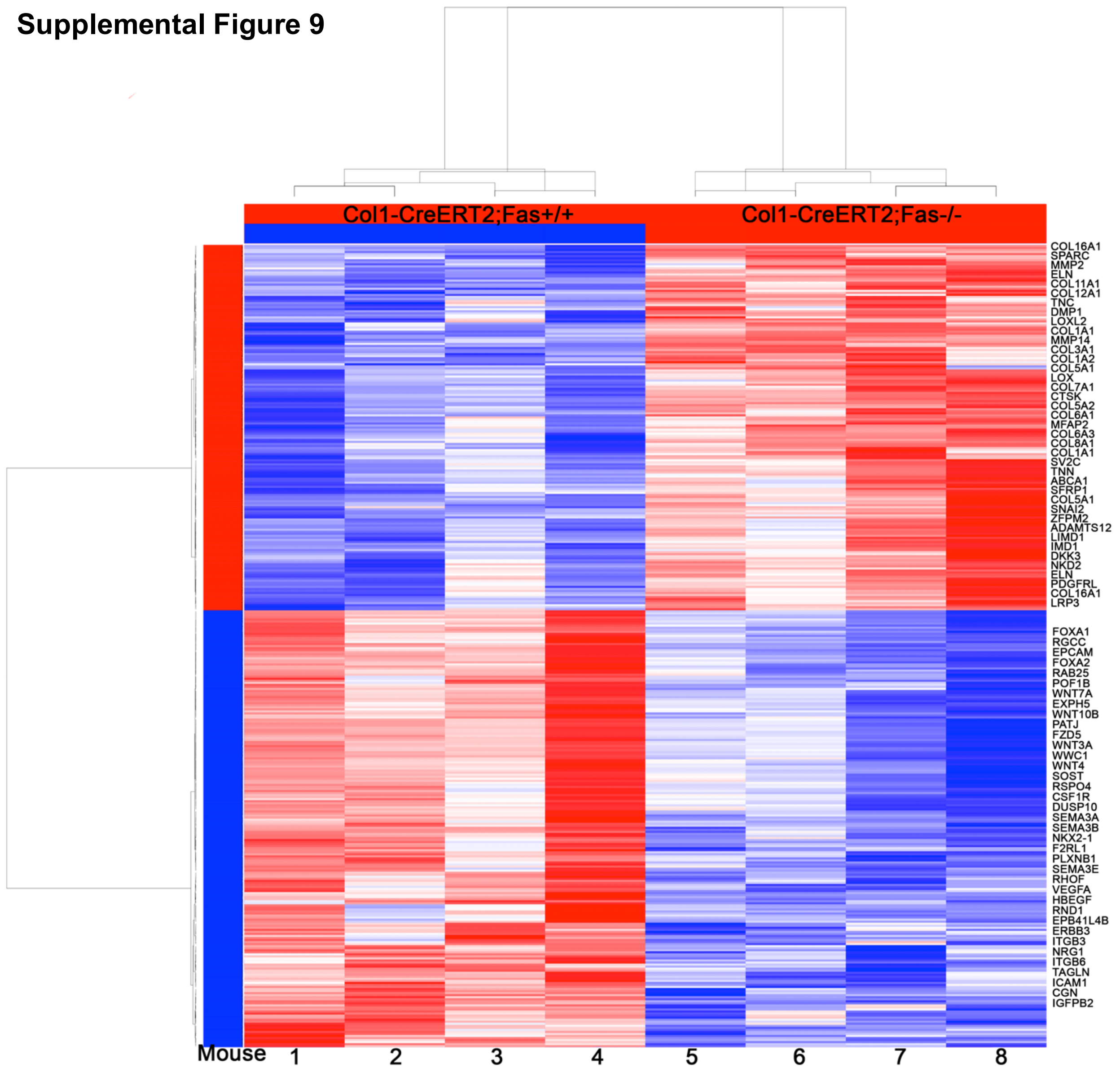

Supplemental Figure 9. Transcriptome expression in bulk sequencing of Lin (-) fibroblasts. Heat maps of normalized signal show transcriptional differences between Lin(-) fibroblasts from Col1-CreERT2;Fas$^{+/+}$ and Col1-CreERT2;Fas$^{-/-}$ mice at six weeks after bleomycin. (n=4/group)

Supplemental Figure 10

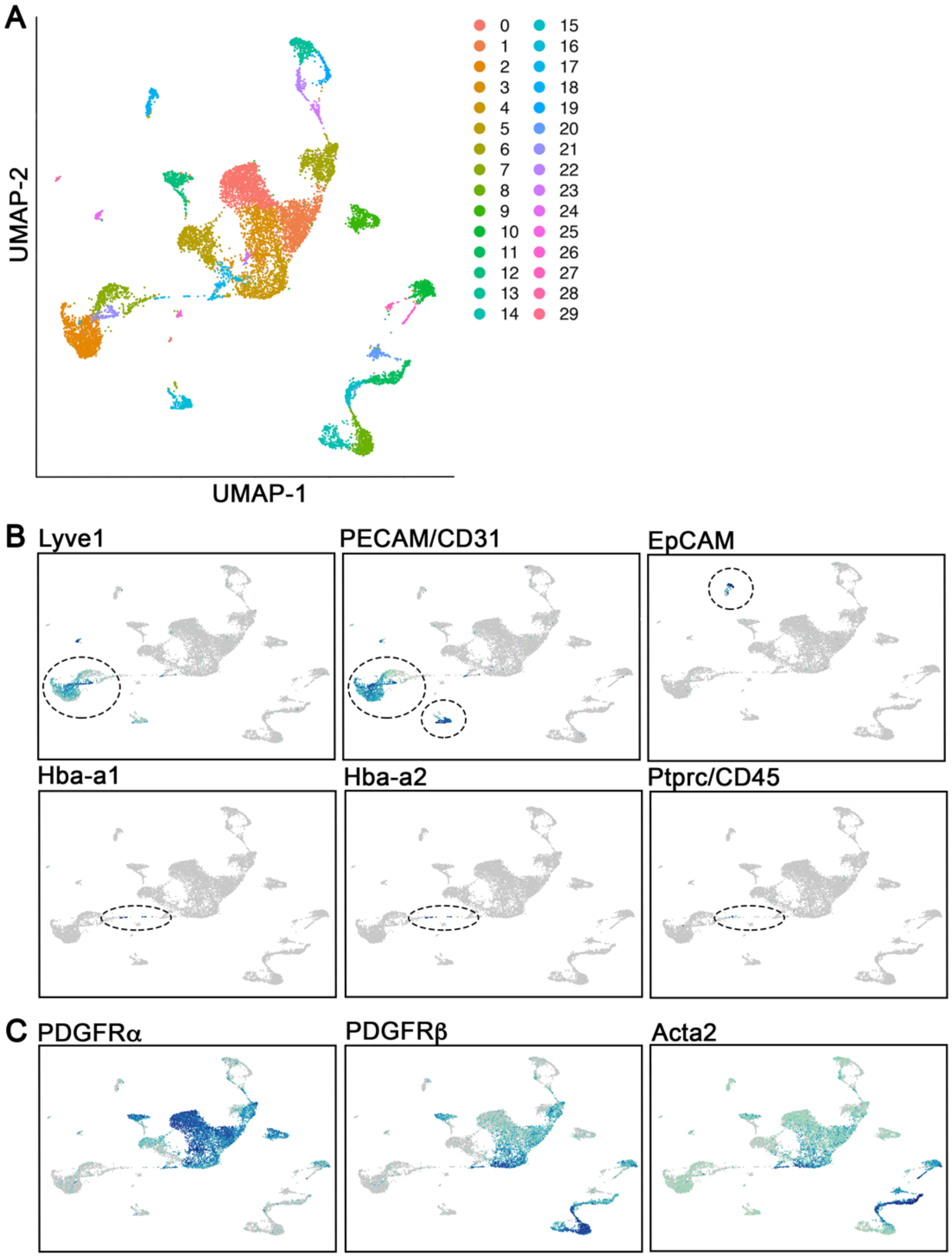

Supplemental Figure 10. Cell clusters identified from Lin(-) sorting by sc-RNA sequencing. (A) UMAP of all samples pre-filtering. (B) UMAP overlays of gene expression identifying cell clusters as non-fibroblasts (C) UMAP overlays of gene expression identifying cell clusters as mesenchymal cells and fibroblasts.

Supplemental Figure 11

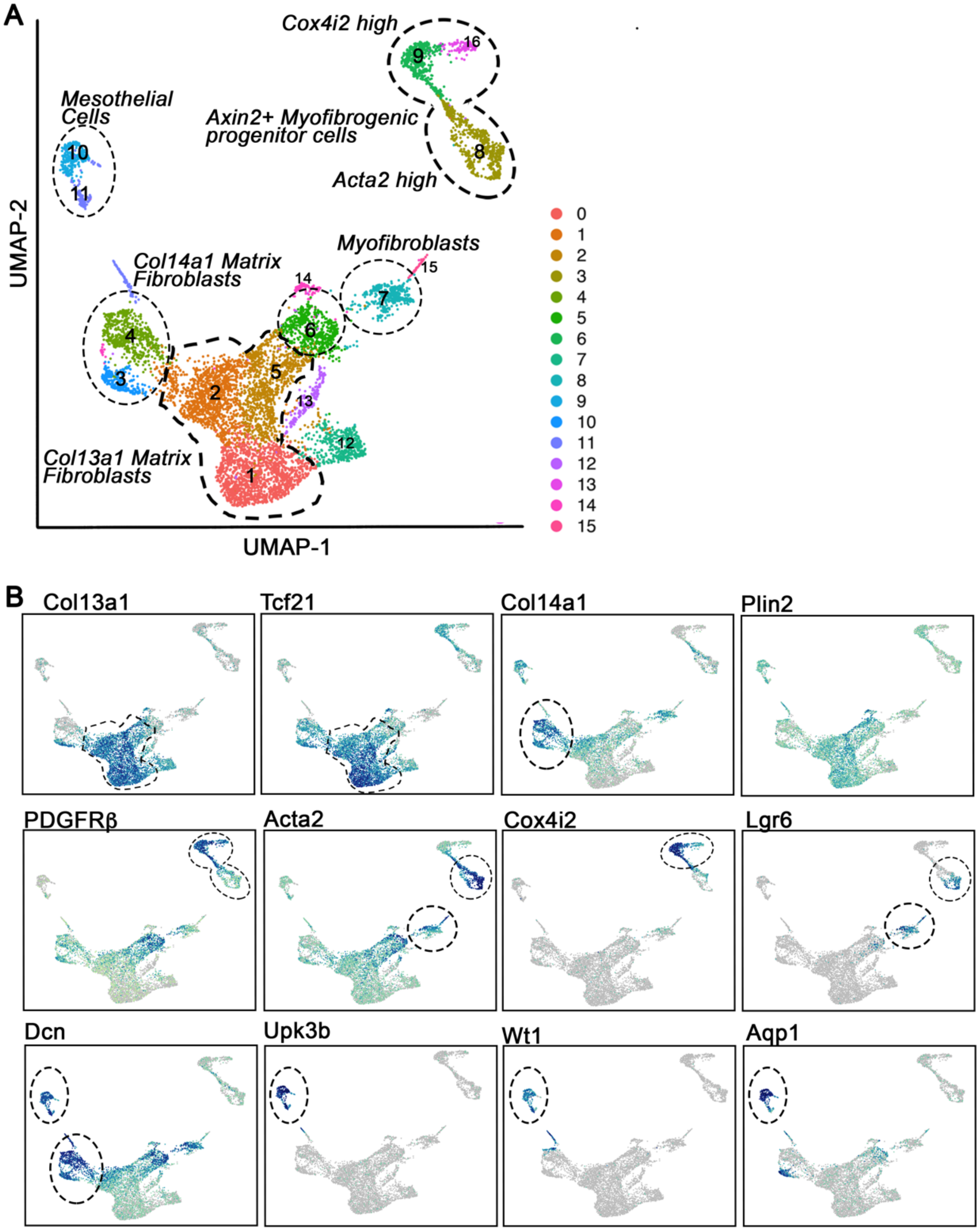

Supplementary Figure 11. Sc-RNA cluster identification based from currently recognized populations. (A) UMAP of all samples with clusters identified/named from the literature (28,29). (B) UMAP overlays of gene expression identifying cell clusters.

Supplemental Figure 12

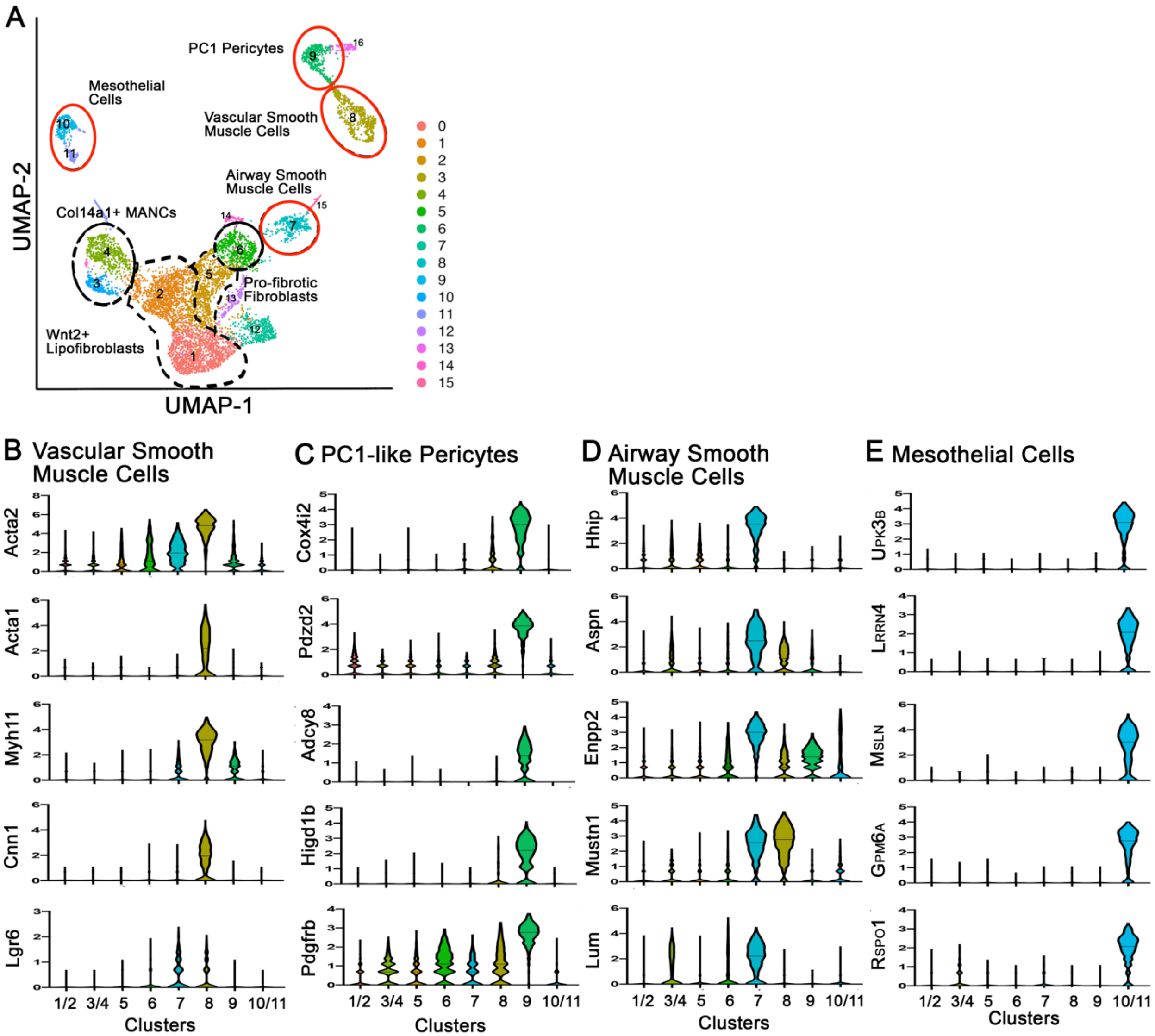

Supplemental Figure 12. Sc-RNA sequencing cluster quality analysis. (A) RNA counts for identified clusters. (B) Feature RNA counts for identified clusters. (C) Percent ribosomal RNA counts for identified clusters. (D) Percent mitochondrial RNA counts for identified clusters. Cluster 12 was only distinguishable from cluster 1 due to low expression of ribosomal RNAs and high expression of mitochondrial RNAs. Cluster 13 was only differentiated due to its high levels of ribosomal RNAs**.** Cluster 15 was only distinguishable from cluster 7 due to high expression of mitochondrial RNAs. Cluster 16 was only distinguishable from cluster 9 due to1low expression of ribosomal RNAs and high expression of mitochondrial RNAs.

Supplemental Figure 13

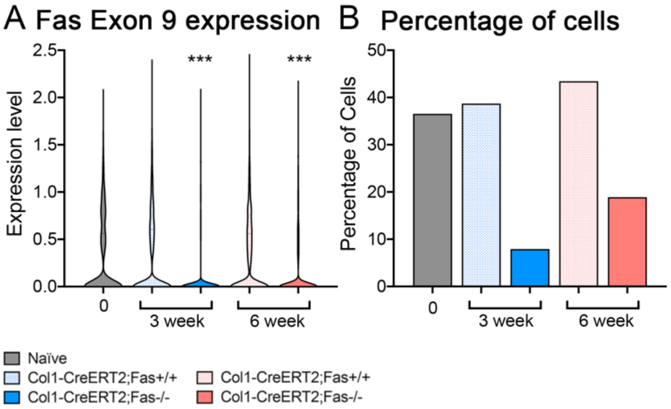

Supplemental Figure 13. Expression of Fas exon 9. (A) Expression of Fas exon 9 in naïve, Fas sufficient and Fas-deficient cells. (B) Percentage of cells expression Fas exon 9 in naïve, Fas sufficient and Fas-deficient cells. Violin plots show median (solid line) and quartiles (dotted line). ***$p<0.001$. 2-tailed *t* test with Welch's correction

Supplemental Figure 14

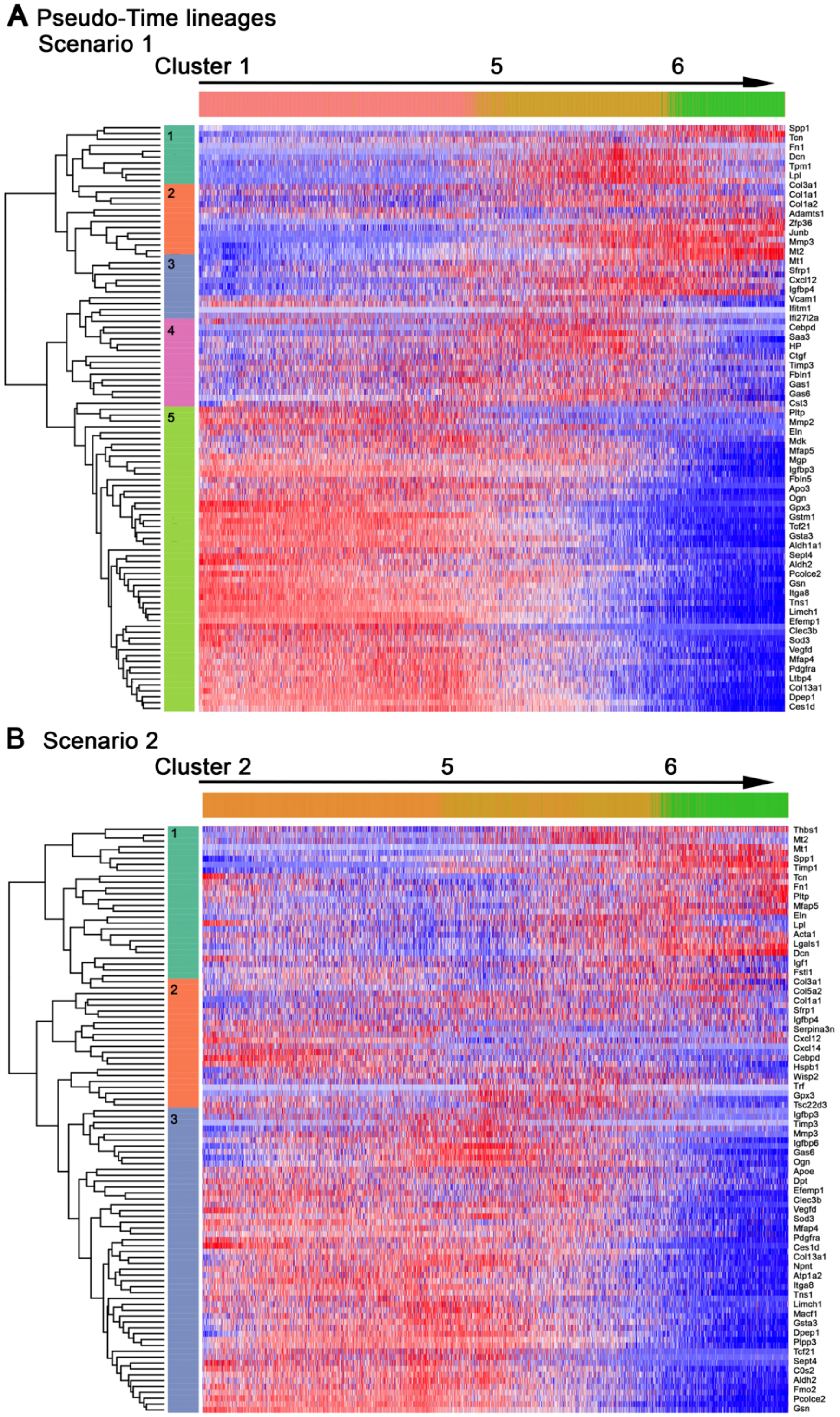

Supplemental Figure 14. Genes associated with pseudotime trajectory analysis. (A) Heatmap shows differentially expressed genes between clusters 1, 5 and 6 over pseudotime scenario 1. (B) Heatmap shows differentially expressed genes between clusters 2, 5 and 6 over pseudotime scenario 2.

Supplemental Figure 15

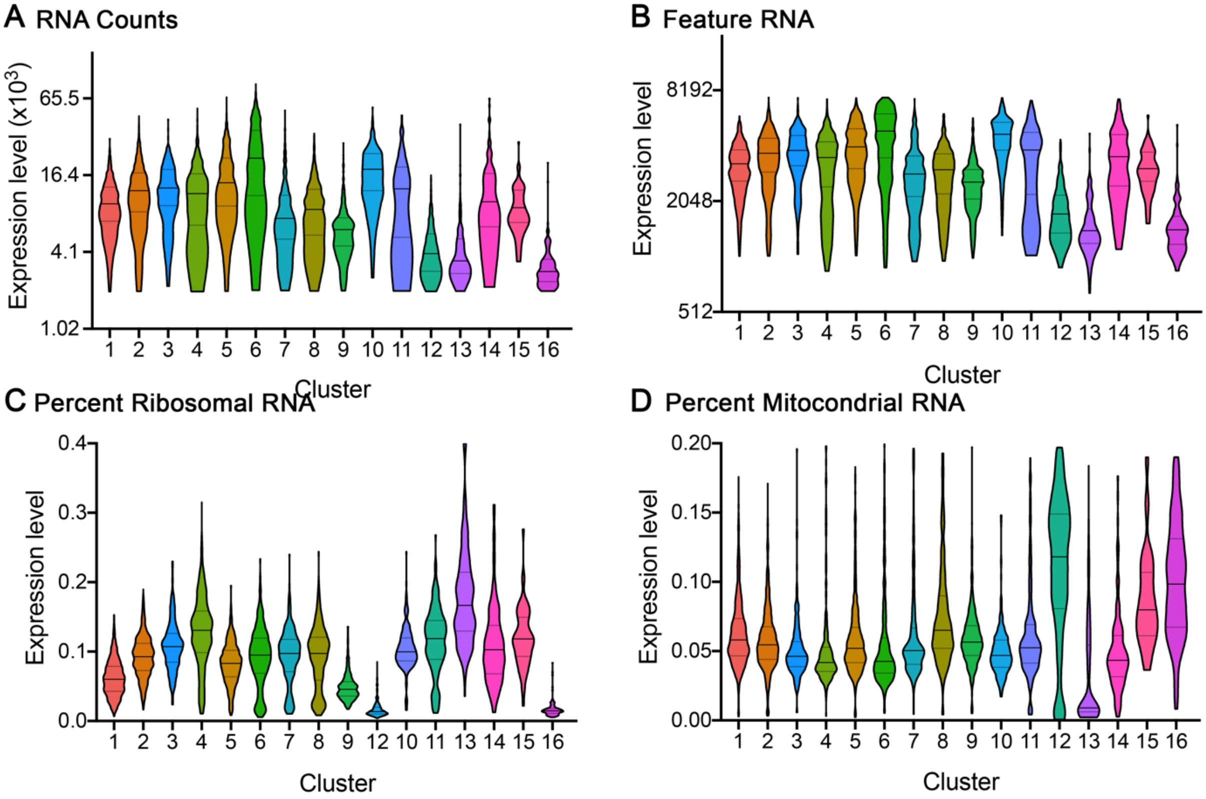

Supplemental Figure 15. Gene expression profile of mesenchymal clusters. (A) UMAP of sc-RNA clusters. (B) Violin plots showing genes highly associated with vascular smooth muscle cells (cluster 8). (C) Violin plots showing genes highly associated with PC1-like pericytes (cluster 9). (D) Violin plots showing genes highly associated with airway smooth muscle cells (cluster7). (E) Violin plots showing genes highly associated with mesothelial cells (clusters 10 and 11). Violin plots show median (solid line) and quartiles (dotted line).

Supplemental Table 1: Lineage markers used for cell population identification

<table>
<tr><th>Lineage</th><th colspan="2">Markers</th><th>Fibroblast subsets</th></tr>
<tr><td>Fibroblasts</td><td colspan="2">Lin-; PDGFRα^{var}</td><td rowspan="4">CD90+
CD26+
CD90- CD26-</td></tr>
<tr><td>Endothelial</td><td>CD31+</td><td rowspan="3">Lin+</td></tr>
<tr><td>Epithelial</td><td>CD326+</td></tr>
<tr><td>Hematopoietic</td><td>CD45+</td></tr>
</table>

Supplemental Table 2: Enrichment pathways from bulk sequencing of Lin- fibroblasts at 6 weeks

Genotype	Enrichment Pathways	Adjusted p value
Col1-CreERT2;Fas-/-	ECM organization	6.32E-13
	Collagen fibril organization	6.59E-09
	Endodermal cell differentiation	1.45E-05
	Platelet-derived growth factor binding	2.76E-05
	ECM receptor interactions	0.00136
	Negative regulation of cell differentiation	0.00618
	Negative regulation of canonical Wnt Signaling	0.1489
Col1-CreERT2;Fas+/+	Regulation of cell-cell adhesion mediated by cadherin	0.00786
	Regulation of cell migration	0.0079
	Epithelial cell development	0.0357
	Hippo signaling	0.0584
	Wnt signaling	0.0622
	Wound healing	0.0699

Supplemental Table 3: Enrichment pathways from single cell sequencing at 6 weeks between Col1-CreERT2;Fas$^{-/-}$ and Col1Cre-ERT2;Fas$^{+/+}$ mice

Cluster	Enrichment Pathways	Adjusted p value
Col13a1 matrix fibroblasts (cluster 1)	Sulfur compound biosynthetic process	0.000952
	Mesonephric tubule development	0.000422
	Actin cytoskeleton reorganization	0.00128
	Positive regulation of vasculature development	0.00153
	Regulation of endothelial cell migration	0.00208
Col13a1 matrix fibroblasts (cluster 2)	Extracellular matrix organization	0.00189
	Extracellular matrix assembly	0.0175
	Endoplasmic reticulum lumen	0.0057
	Elastic fibril formation	0.0072
	Molecules associated with elastic fibers	0.024
	Collagen biosynthesis and modifying enzymes	0.0335
Pro-fibrotic fibroblasts (cluster 5)	Extracellular matrix organization	1.234E-15
	Regulation of cell migration	3.662E-06
	Extracellular matrix disassembly	1.963E-05
	Regulation of smooth muscle cell migration	0.000851
	Cytokine-mediated signaling pathway	0.000901
Pro-fibrotic fibroblast (cluster 6)	Extracellular matrix organization	1.916E-29
	Collagen fibril organization	5.207E-10
	Skeletal system development	7.199E-09
	Endoderm formation	1.496E-07
	Regulation of cell migration	5.711E-07
	Regulation of cell proliferation	5.519E-07
	Negative regulation of apoptotic process	3.599E-06
Col14a1 matrix fibroblasts (cluster 3)	Co-translational protein targeting to membrane	9.16E-20
	Cytosolic ribosome	1.421E-18

	Protein targeting to ER	1.999E-18
	Extracellular matrix organization	9.297E-11
Col14a1 matrix fibroblasts (cluster 4)	SRP-dependent co-translational protein targeting to membrane	6.138E-88
	Co-translational protein targeting to membrane	3.289E-86
	Protein targeting to ER	4.807E-86
	Peptide biosynthetic process	6.899E-63

Supplemental Table 4: Pseudotime trajectory scenario 1 (clusters 1-5-6)

Gene	Phase	Enrichment Pathways	Adjusted p value
Spp1	Early (teal)	ECM-receptor interactions	8.18E-08
Tnc			
Fn1		Focal adhesion	3.4E-06
Dcn			
Tpm1		Protein digestion and absorption	0.000391
Ccdc80			
Lpl		PI3K-Akt signaling pathway	0.000025
Col3a1			
Col1a1			
Col1a2			
Adamts1	Mid (orange, blue, pink)	Lung fibrosis	0.036
Zfp36			
Junb		TNF signaling pathway	0.00191
Id3			
Mmp3		TGF-beta signaling pathway	0.05327
Mt2			
Mt1		Matrix metalloproteinases	0.01727
Cpxm1			
C4b			
Sfrp1			
Cxcl12			
Igfbp4			
Spon2			
Vcam1			
Dpt			
Ifitm1			
Ifi27l2a			
Cebpd			
Saa3			
Hp			
Cp			
Serpina3n			
C3			
Lars2			
Ctgf			
Timp3			
Gm13889			
Fbln1			
Nbl1			
Gas1			
Gas6			
Cst3			
Pltp			
Mmp2			
Eln			
Mdk			
Mfap5			
Mgp			
Igfbp3	Late (green)	Glutathione metabolism	0.00062
Crispld2			
Mat2a		Oxidative stress	0.0024
Adm			

Fbln5		Glycerolipid metabolism	0.011
Adamtsl2			
Mamdc2			
Gyg			
Apoe			
Cdo1			
Ogn			
Gpx3			
Gstm1			
Ppp1r14a			
Tcf21			
Gsta3			
Aldh1a1			
Sept4			
Aldh2			
Selenbp1			
Fmo2			
Mettl7a1			
Inmt			
Pcolce2			
Gsn			
C7			
Pid1			
Dock4			
Fhl1			
Prex2			
Itga8			
Tns1			
Npnt			
Limch1			
Macf1			
Slc43a3			
Efemp1			
Clec3b			
Sod3			
Vegfd			
Mfap4			
Slc7a10			
Scube2			
Pdgfra			
Atp1a2			
Ltbp4			
Col13a1			
Dpep1			
Dpep1			
Plpp3			
Ces1d			
Hsd11b1			

Supplemental Table 5: Psueotime trajectory scenario 2 (clusters 2-5-6)

Gene	Phase	Enrichment Pathways	Adjusted p value
Thbs1	Early (orange)	ECM-receptor interactions	2.03E-05
Mt2			
Mt1		Focal adhesion formation	2.66E-05
Spp1			
Timp1		PI3K-Akt signaling pathway	0.000533
Tnc			
Fn1		Protein digestion and absorption	0.000403
Pltp			
Mdk			
Mfap5			
Pmepa1			
Eln			
Lpl			
Serpine2			
Mgp			
Acta2			
Crip1			
Lgals1			
Dcn			
Aebp1			
Igf1			
Fstl1			
Ccdc80			
Col3a1			
Col5a2			
Col1a1			
Sfrp1	Mid (blue)	Cholesterol metabolism	0.05256
Igfbp4			
Saa3		VEGF signaling pathway	0.06193
Hp			
Serpina3n		Glutathione metabolism	0.06812
Cxcl12			
Gm13889			
Cxcl14			
Angptl4			
Ifi27l2a			
Cebpd			
Rgs2			
Hspb1			
Zfp36			
Id3			
Txnip			
Adm			
Wisp2			
Trf			
Gyg			
Cdo1			
Klf2	Late (pink)	Matrix metalloproteinases	0.08718
Tsc22d3			
Igfbp3		Pathways affecting insulin-like growth factor (IGF1)-Akt signaling	0.08294
Timp3			

Mmp3			
Spon2			
Igfbp6			
Nbl1			
Cpxm1			
C4b			
C3			
Gas6			
Ogn			
Apoe			
Cst3			
Dpt			
Efemp1			
Clec3b			
Mamdc2			
Gpc3			
Vegfd			
Sod3			
Mfap4			
Slc7a10			
Scube2			
Pdgfra			
Ces1d			
Aldh1a1			
Col13a1			
Hsd11b1			
Npnt			
Atp1a2			
Prex2			
Itga8			
Tns1			
Limch1			
Macf1			
C7			
Gsta3			
Dpep1			
Plpp3			
Ppp1r14a			
Tcf21			
Sept4			
G0s2			
Aldh2			
Mettl7a1			
Selenbp1			
Fmo2			
Pcolce2			
Inmt			
Gsn			
